# A Facile and Catalyst-Free Microwave-Promoted Multicomponent Reaction for the Synthesis of Functionalised 1,4-Dihydropyridines With Superb Selectivity and Yields

**DOI:** 10.3389/fchem.2021.638832

**Published:** 2021-03-31

**Authors:** Nagaraju Kerru, Suresh Maddila, Sreekantha B. Jonnalagadda

**Affiliations:** ^1^Department of Chemistry, GITAM School of Science, Gandhi Institute of Technology and Management (GITAM) University, Bengaluru, India; ^2^School of Chemistry and Physics, University of KwaZulu-Natal, Durban, South Africa; ^3^Department of Chemistry, GITAM Institute of Sciences, Gandhi Institute of Technology and Management (GITAM) University, Visakhapatnam, India

**Keywords:** microwave irradiation, multi-component reaction, aqueous medium, 1, 4-dihydropyridine, one-pot method

## Abstract

We report a highly efficient green protocol for developing a novel library of 1,2,4-triazole-tagged 1,4-dihydropyridine analogs through the one-pot process from the four-component fusion of the 1*H*-1,2,4-triazol-3-amine with different chosen aldehydes, diethyl acetylenedicarboxylate, and active methylene compounds in a water medium under microwave irradiation and catalyst-free conditions. Excellent yields (94–97%) of the target products were achieved with high selectivity with a short reaction time (<12 min) at room temperature. The structures of the synthesized pyrimidine analogs were established by NMR and HRMS spectroscopic analysis. Simple workup, impressive yields, no column chromatography, green solvent, rapid reaction, and excellent functional group tolerance are the benefits of this protocol.

**Graphical Abstract:**
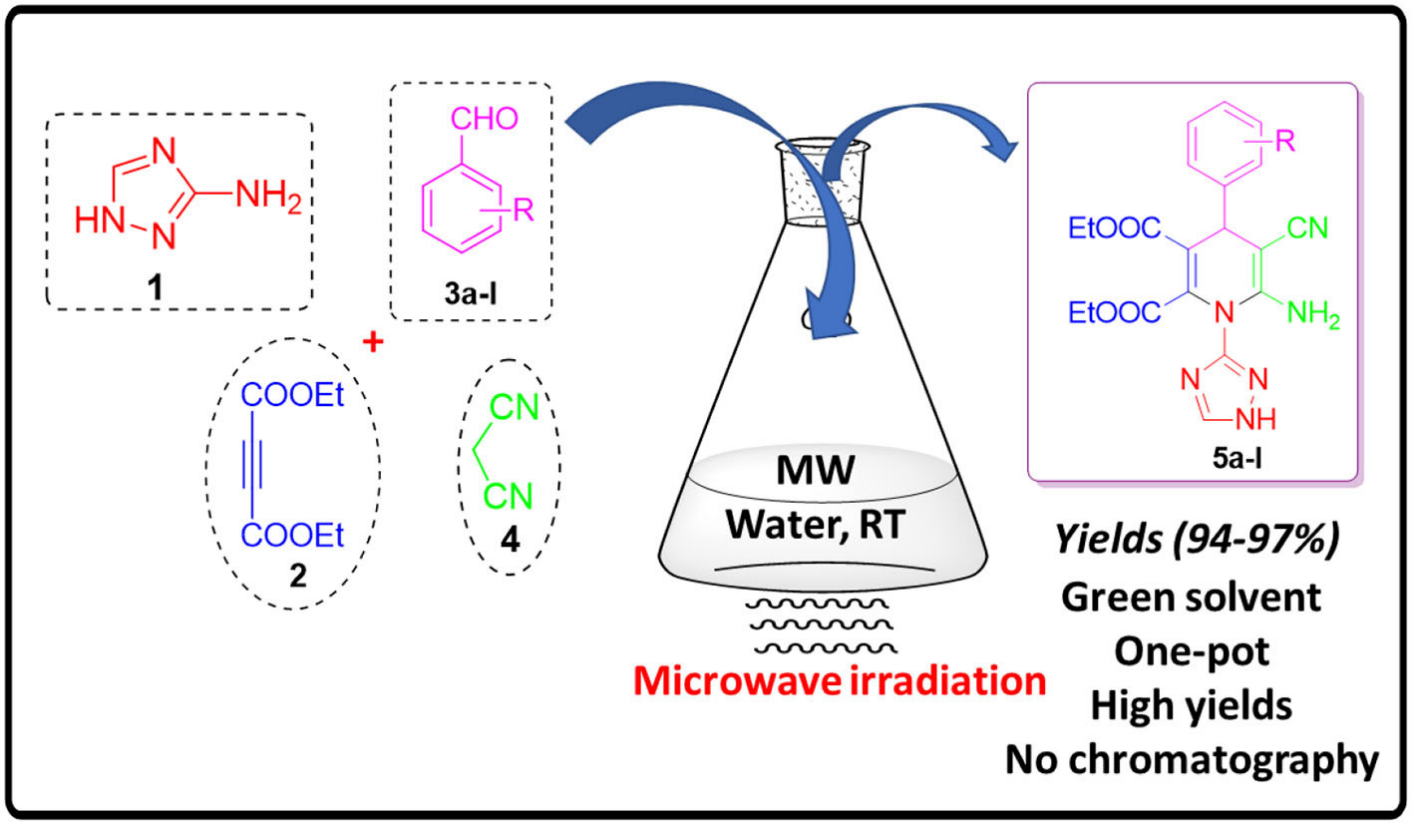
Synthesis of Fictionalized 1,4-Dihydropyridines Under Microwave Irradiation and Aqueous Conditions.

## Introduction

The green chemistry approach employing non-hazardous chemicals and eco-friendly reaction conditions is most motivating in preparing broadly used and pharmacologically significant organic compounds (Kerru et al., 2019a,b). The microwave irradiation (MW) technique mostly applies environmentally benign green technology in heating to speed up the organic reactions for value-added conversions. The MW-assisted organic synthesis has become a powerful tool and an excellent non-conventional approach in the green synthetic methodologies in modern drug discovery programs (Wathey et al., 2002; Mavandadi and Pilotti, 2006). MW induces the growth of oscillation excitation and mass transference in a microwave environment, which produces vigorous heating in reaction vessels, an alternative energy source of chemical reaction for enhanced reaction rate (Polshettiwar and Varma, 2008). Furthermore, the MW technique has attracted considerable interest in organic synthesis due to the higher selectivity, improved product yield, and atom economy, which reduce the by-product generation compared to conventional thermal heating techniques (Khumalo et al., 2019). Therefore, MW-mediated organic synthesis is the preferred green method over unconventional to classical thermal processes for the rapid synthesis of a series of bioactive heterocyclic compounds (Diaz-Ortiz et al., 2019). In recent years, multicomponent reactions (MCR) have also gained eminence as ecofriendly green procedures in synthetic organic chemistry (Kerru et al., 2020a,b). The one-pot processes afford a high atom economy, high functional group tolerance, avoidance of the separation and purification methods, and minimizing the chemical waste (Kerru et al., 2020c,d). Thus, the MCR strategy is an economical approach for the synthesis of libraries of heterocyclic molecules for medicinal chemistry benefits (Maddila et al., 2020a).

Heterocyclic moieties are valuable scaffolds in the drug innovation program and exploring potential biological applications (Maddila et al., 2016; Kerru et al., 2020e). Among the *N*-heterocyclic molecules, the 1,4-dihydropyridine skeleton has gained extra significance due to a broad array of pharmacological activities such as calcium channel blocking, antidyslipidemic, antioxidant, antidiabetic, antibacterial, and antimycobacterial activities, and it has also shown to have an effect on Alzheimer's disease (Kumar et al., 2010; Sirisha et al., 2011; Niaz et al., 2015; Schaller et al., 2018; Malek et al., 2019). Recent literature reports show the increasing approval toward the synthesis of 1,4-dihydropyridine moieties through MCR strategy. Some multicomponent reactions have been reported in the literature for the synthesis of different 1,4-dihydropyridine derivatives, which employed catalysts, including sulfamic acid, Fe_3_O_4_/KCC-1/BPAT, Gd(OTf)_3_, aminated CNTs, hydromagnesite, and nano-ZrO_2_-SO_3_H (Rajesh et al., 2013; Amoozadeh et al., 2016; Sadeghzadeh, 2016; Sheik Mansoor et al., 2017; Mahinpour et al., 2018; da Costa Cabrera et al., 2019). In the recent past, different procedures for 1,4-dihydropyridines in good yields have been reported to employ heterogeneous catalysts, such as V_2_O_5_/ZrO_2_ (Bhaskaruni et al., 2018), USY-zeolit (Alponti et al., 2021), and Fe_3_O_4_@SiO_2_ (Saffarian et al., 2021). Davarpanah et al. (2019) reported nicotinic acid as a catalyst for the Hantzsch synthesis dimedone and different aldehydes under solvent-free conditions obtaining the desired products in high yields. However, eco-friendly and sustainable protocols are still in demand to synthesize 1,4-dihydropyridine derivatives under green conditions.

Our continuous strive for green methodologies by applying the MCR approach, we earlier have been reported various synthetic procedures (Kerru et al., 2019c, 2020f,g,h; Maddila et al., 2020b). Herein, we describe the synthesis of 12 novel, functionalised 1,2,4-triazole-agged 1,4-dihydropyridine scaffolds through a one-pot process. A four-component reaction between 3-amino-1,2,4-triazole, diethyl acetylenedicarboxylate, malononitrile, and various chosen aldehydes in water under microwave irradiation at room temperature was utilized.

## Experimental Section

### General Procedure for the Synthesis of 1,2,4-Triazole-1,4-Dihydropyridine (5a-l) Under Microwave

A solution of 1*H*-1,2,4-triazol-3-amine (**1**, 0.1 mmol), diethyl acetylenedicarboxylate (**2**, 0.1 mmol), chosen aldehydes (**3a-l**, 0.1 mmol), and malononitrile (**4**, 0.1 mmol) in water solvent (6 mL) were added in a 100 mL volume shielded combination vessel. The reaction mixture was MW aided by exploitation microwave irradiation power (150 W) at room temperature for 10–12 min. The reaction progress was monitored by TLC (Hexane: Ethyl acetate; 70:30). After the completion of the reaction, the reaction mixture was transferred to the beaker. The synthesized solid product was filtered by vacuum before being the product was recrystalised with hot ethanol to offer the corresponding pure product. The structural elucidation of all the novel compounds was accomplished by different spectroscopic methods (^1^H NMR, ^13^C NMR, and HRMS).

### Diethyl 6-Amino-5-Cyano-4-(4-Methoxyphenyl)-1-(1H-1,2,4-Triazol-3-yl)-1,4-Dihydropyridine-2,3-Dicarboxylate (5a)

White solid; ^1^H NMR (400 MHz, CDCl_3_) δ 8.36 (s, 1H, triazole-CH), 7.93 (d, *J* = 8.9 Hz, 2H, Ar-H), 7.68 (s, 2H, NH_2_), 7.04 (d, *J* = 8.9 Hz, 2H, Ar-H), 6.55 (s, 1H, NH), 4.96 (s, 1H, CH), 4.38 (q, *J* = 7.6 Hz, 4H, 2 × CH_2_), 3.94 (s, 3H, OCH_3_), 2.34 (t, *J* = 7.4 Hz, 6H, 2 × CH_3_); ^13^C NMR (100 MHz, CDCl_3_) δ 174.38, 170.30, 164.85, 158.92, 146.80, 141.43, 133.49, 128.41, 124.04, 115.16, 114.46, 113.38, 78.87, 64.11, 55.84, 34.64, 13.81; HRMS of [C_21_H_22_N_6_O_5_ + 1]^+^ (*m/z*) 439.1517; Calcd: 439.1509.

## Results and Discussion

To examine the solvent effect and reaction conditions for the synthesis of 1,2,4-triazole tagged 1,4-dihydropyridine **4a** on the reaction rate and yield of the desired product and results are illustrated in Tables 1, 2. Initially, the typical reaction between the equimolar mixture of 3-amino-1,2,4-triazole (**1**), diethyl acetylene dicarboxylate (**2**), *para*-methoxy benzaldehyde (**3a**), and malononitrile (**4**) was performed at room temperature (RT) under solvent-free conditions. Both microwave (1 h) and classical heating (2.5 h) reactions gave a low yield of the product (Table 1, entry 1). We further investigated the solvent's impact on the product formation by using different polar-aprotic (CH_3_CN, DCM, and THF) and polar-protic (AcOH, H_2_O, MeOH, and EtOH) solvents. The polar-aprotic solvents gave the low yields of the desired product at RT under both conditions (Table 1, entries 2–4). The polar-protic solvents offered improved yields of the target product in shorter reaction time observed with both the methods (Table 1, entries 5–8). Among the solvents, water provided the best yield (96%) with a short reaction time (10 min) under microwave irradiation conditions (Table 1, entry 6). In an aqueous medium, the hydrogen bond development with starting substrates accelerates the reaction rate.

**Table 1:** Optimized effect of solvent under catalyst-free for the 1,2,4-triazol-1,4-dihydropyridine (**4a**) formation^a^.

**S.No**	**Medium**	**Microwave**		**Conventional**	
		**Time in min**	**Yield (%)^b^**	**Time in min**	**Yield (%)^b^**
1	Solvent-free	60	18	150	19
2	CH_3_CN	60	36	120	24
3	DCM	60	22	120	21
4	THF	60	26	120	18
5	AcOH	30	68	120	56
6	H_2_O	10	96	90	81
7	MeOH	10	82	90	72
8	EtOH	10	87	90	79

a *The reaction was performed with 1H-1,2,4-triazol-3-amine (**1**, 0.1 mmol), diethyl acetylenedicarboxylate (**2**, 0.1 mol), para-methoxy benzaldehyde (**3a**, 0.1 mmol), malononitrile (**4**, 0.1 mmol) and solvent (6.0 mL) at room temperature*.

b *Isolated yields*.

**Table 2:** Examined the efficiency of catalyst for the 1,2,4-triazol-1,4-dihydropyridine (**4a**) formation^a^.

**S.No**	**Catalyst**	**Microwave**		**Conventional**	
		**Time (min)**	**Yield (%)^b^**	**Time (min)**	**Yield (%)^b^**
1	Catalyst free	10	96	90	81
2	NaOH	10	78	90	78
3	KOH	10	77	90	76
4	Cs_2_CO_3_	10	82	90	68
5	K_2_CO_3_	10	73	90	79
6	Et_3_N	10	92	90	83
7	NH_4_OAc	10	94	90	69

a *Reaction conditions: 1H-1,2,4-triazol-3-amine (**1**, 0.1 mmol), diethyl acetylenedicarboxylate (**2**, 0.1 mol), para-methoxy benzaldehyde (**3a**, 0.1 mmol), malononitrile (**4**, 0.1 mmol), catalyst (10 mol%) and water (6.0 mL) at RT*.

b *Isolated yields*.

Moreover, water is eco-friendly and inexpensive than the other organic solvents. With water as the solvent, the classical heating reaction required a longer time (1.5 h) and gave 81% of the target product (Table 1, entry 6). The overall results revealed that the microwave irradiation protocol provided high yields of the corresponding products in less time than the classical heating. Thus, water was shown to be the best medium for product yield and reaction time for synthesizing 1,2,4-triazole-tagged 1,4-dihydropyridine scaffolds under microwave irradiation at RT.

Additionally, we investigated the catalysts' ability to enhance the yield. Runs were conducted with NaOH, KOH, Cs_2_CO_3_, K_2_CO_3_, trimethylamine, and ammonium acetate as a catalyst under otherwise similar conditions (Table 2). Lesser yields were detected under MW (73–94%) for 10 min and under classical heating for 90 min (68–83%) (Table 2, entries 2–7) as compared to the catalyst-free condition (Table 2, entry 1). The reaction without catalyst provided superior yields under both conditions.

**Scheme 1:**
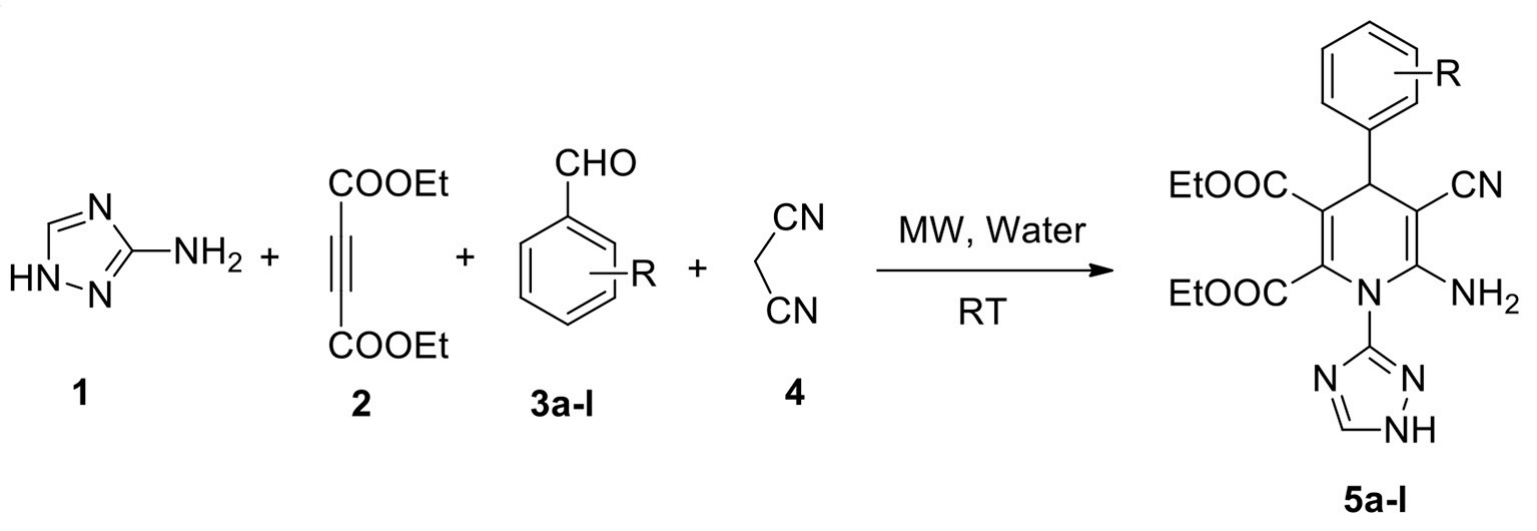
Synthesis of 1,2,4-triazole-1,4-dihydropyridine derivatives (**5a-l**).

Under these optimized conditions (water and catalyst-free), we explored the scope and efficacy of this procedure, engaging different aldehyde substrates (**3a-l**) and C-H active compounds, (**4**) acetylenedicarboxylate (**2**) and reacting with 3-amino-1,2,4-triazole (**1**) under MW conditions (Scheme 1). A total of 12 of the 1,2,4-triazole-linked 1,4-dihydropyridine analogs (**5a-l**) were synthesized in excellent yields (94–97%) within a reaction time of <12 min (Table 3), and all were novel compounds. We observed that the different electron-deficient and electron-rich functional groups on the aldehydes' phenyl moiety were well-tolerated and performed effectively, offering the desired product's excellent yields.

**Table 3:** Synthesis of 1,2,4-triazole tagged 1,4-dihydropyridine scaffolds **4a-l**^a^.

**S.No**	**R**	**Compound**	**Time (min)**	**Yield (%)^b^**	**M.p./^**°**^C**
1	4-OCH_3_	**4a**	10	95	210–212
2	4-Cl	**4b**	10	96	206–208
3	4-Br	**4c**	10	95	214–216
4	3,4-di-OCH_3_	**4d**	9	95	202–204
5	4-SCH_3_	**4e**	11	94	196–198
6	4-CH_2_CH_3_	**4f**	9	94	201–203
7	4-N(CH_3_)_2_	**4g**	11	97	221–223
8	3-Br	**4h**	12	97	199–201
9	3-OCH_3_	**4i**	10	96	194–196
10	2,4,5-tri-OCH_3_	**4j**	11	95	203–205
11	2-OCH_3_	**4k**	9	96	202–204
12	4-CH_3_	**4l**	12	97	209–211

a *Reaction conditions: 1H-1,2,4-triazol-3-amine (**1**, 0.1 mmol), diethyl acetylenedicarboxylate (**2**, 0.1 mol), para-methoxy benzaldehyde (**3a-l**, 0.1 mmol), malononitrile (**4**, 0.1 mmol), and water (6.0 mL) under microwave irradiation at RT*.

b *Isolated yields*.

All the synthesized scaffold structures were elucidated and confirmed by ^1^H and ^13^C NMR and HRMS spectroscopic analysis (Supplementary Material). For example, the synthesized compound **5a** exhibited two prominent signals at δ 4.96 and δ 7.68 ppm due to the CH and NH_2_ protons of the 1,4-dihydropyridine moiety. The quartet and triplet peaks appeared at δ 4.38 and δ 2.34 ppm attributed to the acetate (COOCH_2_CH_3_) group protons. The two singlet signals at δ 6.55 and δ 8.36 ppm are due to the NH and CH protons of the 1,2,4-triazole ring moiety. Another singlet was appeared at δ 3.94 ppm belongs to the three protons of the methoxy group (OCH_3_) on the phenyl ring. The other residual phenyl ring protons appeared at their corresponding aromatic positions in the ^1^H NMR spectrum. The acetate moiety's carbonyl carbon exhibited at δ 174.38 and 170.30 ppm in the ^13^C NMR spectrum. The HRMS spectrum further confirmed the formation of the condensation product 5a with the molecular-ion peak (M+H) at *m/z* 439.1517. All the synthesized derivatives were established as the 1,2,4-triazole-linked 1,4-dihydropyridine analogs based on the structural characterization data.

Table 4 illustrates the comparison of the results from the proposed green method and literature reported protocols in terms of experimental conditions, reaction time, and yield. An observation of the table's data indicates that eco-friendly solvent water provides superior results under microwave irradiation at RT in all respects compared to the reported methods. Thus, the MW method offers higher yields, excellent selectivity, simple workup, and a rapid reaction under catalyst-free green solvent conditions.

**Table 4:** Comparison of the current reported procedure with previously described methods for the synthesis of 1,4-dihydropyridines.

**S.No**	**Catalyst**	**Reaction conditions**	**Time (h)**	**Yield(%)^**Ref**^**
1	Sulfamic acid	Reflux/MeOH	24	47–92 ^(*daCostaCabreraetal*.,2019)^
2	Fe_3_O_4_/KCC-1/BPAT	Reflux/Water	4	79–88 ^(*Sadeghzadeh*,2016)^
3	Gd(OTf)_3_	RT/Ethanol	6	82–89 ^(*SheikMansooretal*.,2017)^
4	Aminated CNTs	Reflux/ethanol	6	80–96 ^(*Mahinpouretal*.,2018)^
5	Hydromagnesite	90°C/Water	0.75	80–98 ^(*Rajeshetal*.,2013)^
6	Nano-ZrO_2_-SO_3_H	80°C/solvent-free	1	84–93 ^(*Amoozadehetal*.,2016)^
7	Catalyst-free	Microwave/RT/Water	<12 min	94–97 (This work)

**Scheme 2:**
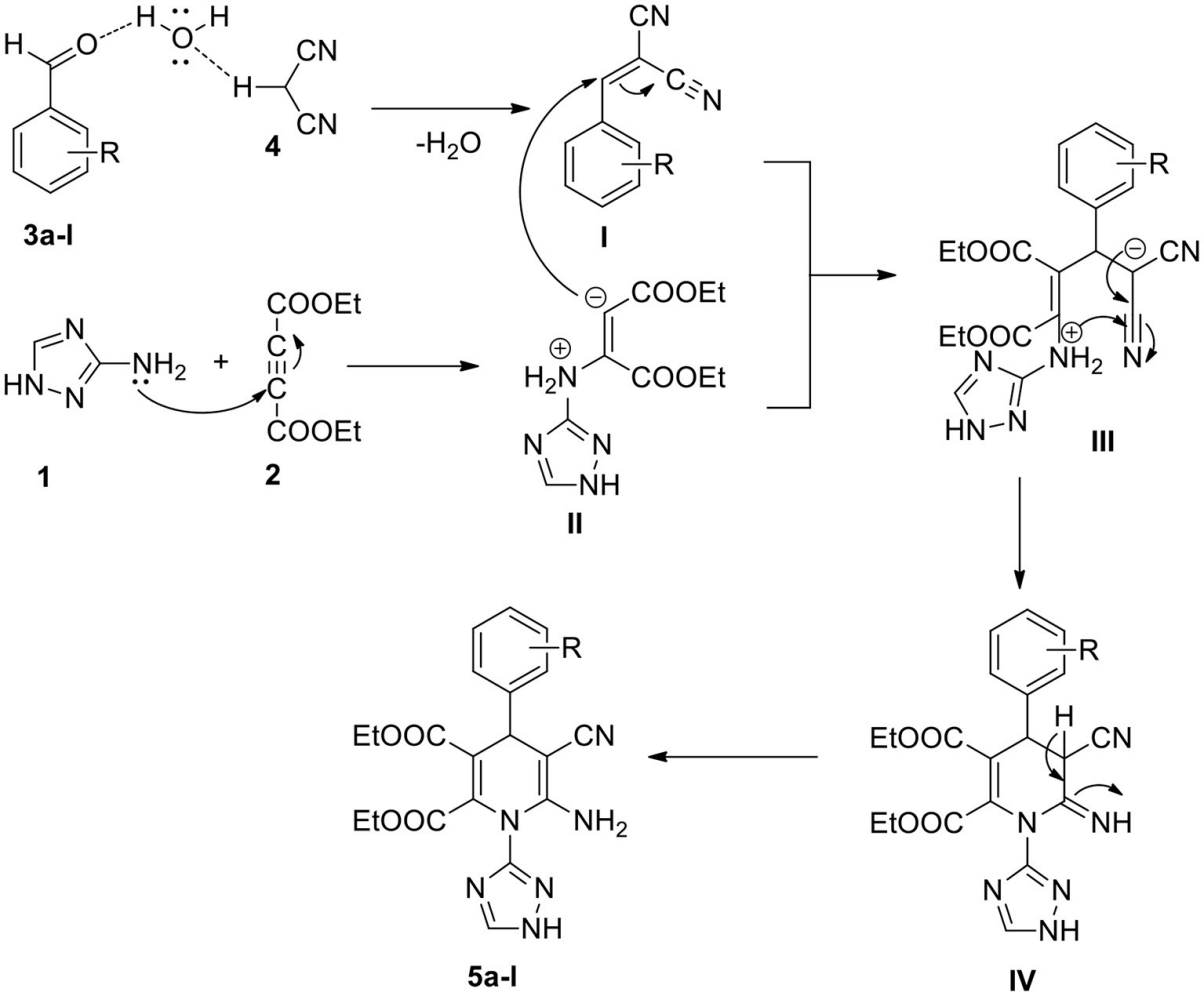
The possible mechanism for the synthesis of 1,2,4-triazole-1,4-dihydropyridines.

A probable mechanism to synthesize 1,2,4-triazole-linked 1,4-dihydropyridine analogs through a one-pot process is proposed (Scheme 2). Water plays a significant part in this conversion. The electrophilicity of the aldehyde substrate (**3a-l**) carbonyl carbon enhances through the hydrogen bond between the water and carbonyl group (Ramesh and Lalitha, 2016; Kerru et al., 2020i). Simultaneously, the acidic hydrogen of malononitrile (**4**) hydrogen bonds with the oxygen of the H_2_O molecule. Then, the Knoevenagel condensation product (**I**) forms by the elimination of water molecules. Subsequently, 1,2,4-triazole-amine (**1**) reacts with diethyl acetylenedicarboxylate (**2**), resulting in the formation of the enolate intermediate (**II**). Then, through the Michael addition, the intermediate (**I**) reacts with intermediate (**II**), generating the transient intermediate (**III**) (Maddila et al., 2019). The intermediate (**III**) undergoes intramolecular cyclisation (**IV**) followed by tautomerisation, finally leading to the generation of the target compound, 1,2,4-triazole-linked 1,4-dihydropyridine derivative (**5a-l**).

## Conclusion

We described the procedure for synthesizing 12 novel biologically imperative 1,2,4-triazole-tagged 1,4-dihydropyridine analogs with excellent yields (94**–**97%) under microwave irradiation conditions. The one-pot reaction between the 3-amino-1,2,4-triazole, diethyl acetylenedicarboxylate, malononitrile, and various selected aldehydes was effectively accomplished in a water medium at RT in <12 min reaction time. The structural elucidation of the synthesized derivatives was achieved by HRMS, ^1^H, and ^13^C NMR spectral analysis. The method's various benefits are catalyst-free and show swift reactions, high selectivity, excellent yields, green solvents, and avoidance of column chromatography and hazardous reagents.

## Data Availability Statement

The original contributions presented in the study are included in the article/Supplementary Material, further inquiries can be directed to the corresponding author.

## Author Contributions

NK performed experimental studies and wrote the original draft. SM performed validation and spectral characterization of synthesized molecules. SJ designed project planning, proofreading, and editing. All authors contributed to the article and approved the submitted version.

## Conflict of Interest

The authors declare that the research was conducted in the absence of any commercial or financial relationships that could be construed as a potential conflict of interest.
