# Supplementary material for: A Facile and Catalyst-Free Microwave-Promoted Multicomponent Reaction for the Synthesis of Functionalised 1,4-Dihydropyridines With Superb Selectivity and Yields
Source: Front Chem. 2021 Mar 31;9:638832. doi: 10.3389/fchem.2021.638832 (PMC8044462; doi:10.3389/fchem.2021.638832)
Supplement: Supplementary file 1 [file Data_Sheet_1.docx]

Supplementary Material

# Supplementary Data

# *Materials and methods*

All chemicals and reagents were ordered analytical grade and were employed without purification. NMR analysis was obtained by Bruker AMX 400 MHz NMR (399.99 MHz for ^1^H and 100.42 MHz for ^13^C) spectrometer and TMS operated as the internal standard in CDCl_3_ (^1^H; 7.26 ppm and ^13^C; 77.3 ppm) and DMSO-*d_6_* (^1^H; 2.50 ppm and ^13^C; 39.51 ppm) as a solvent. Melting points of synthesized analogs were calculated in an electrothermal melting point apparatus (Electrothermal IA9100) applying a wrapped capillary tube and are uncorrected. TLC aluminum plates coated with silica gel (Merck Kieselgel 60 F254) and chemical shift values were described in *δ* (ppm). Microwave-supported reaction was conducted in a CEM-908010, 300-W bench mate model laboratory MW reactor. The MW power was 150 W for the reaction at 80 °C with high stirring. High-resolution mass data were acquired on a Bruker micro TOF-Q II ESI, it is operating at ambient temperature.

***General procedure for the synthesis of 1,2,4-triazole-1,4-dihydropyridine*** *(****5a-l****)* ***under microwave***

A solution of 1*H*-1,2,4-triazol-3-amine (**1**, 0.1 mmol), diethyl acetylenedicarboxylate (**2**, 0.1 mmol), chosen aldehydes (**3a-l**, 0.1 mmol) and malononitrile (**4,** 0.1 mmol) in water solvent (6 mL) were added in a 100 mL volume shielded combination vessel. The reaction mixture was MW aided by exploitation microwave irradiation power (150 W) at room temperature for 10-12 min. The reaction progress was monitored by TLC (Hexane:Ethyl acetate; 70:30). After the completion of the reaction, the reaction mixture was transferred to the beaker. The synthesized solid product was filtered by vacuum. Further, the recrystallization of the product with hot ethanol, to offer the corresponding pure product. The structural elucidation of all the novel compounds is interpreted by different spectroscopic methods (^1^H NMR, ^13^C NMR and HRMS).

*Diethyl 6-amino-5-cyano-4-(4-methoxyphenyl)-1-(1H-1,2,4-triazol-3-yl)-1,4-dihydropyridine-2,3-dicarboxylate* (**5a**)

White solid; ^1^H NMR (400 MHz, CDCl_3_) δ 8.36 (s, 1H, triazole-CH), 7.93 (d, *J* = 8.9 Hz, 2H, Ar-H), 7.68 (s, 2H, NH_2_), 7.04 (d, *J* = 8.9 Hz, 2H, Ar-H), 6.55 (s, 1H, NH), 4.96 (s, 1H, CH), 4.38 (q, *J* = 7.6 Hz, 4H, 2×CH_2_), 3.94 (s, 3H, OCH_3_), 2.34 (t, *J* = 7.4 Hz, 6H, 2×CH_3_); ^13^C NMR (100 MHz, CDCl_3_) δ 174.38, 170.30, 164.85, 158.92, 146.80, 141.43, 133.49, 128.41, 124.04, 115.16, 114.46, 113.38, 78.87, 64.11, 55.84, 34.64, 13.81; HRMS of [C_21_H_22_N_6_O_5_ + 1]^+^ (*m/z*) 439.1517; Calcd: 439.1509.

*Diethyl 6-amino-4-(4-chlorophenyl)-5-cyano-1-(1H-1,2,4-triazol-3-yl)-1,4-dihydropyridine-2,3-dicarboxylate* (**5b**)

Cream white solid; ^1^H NMR (400 MHz, CDCl_3_) δ 8.49 (s, 1H, triazole-CH), 7.89 (d, *J* = 8.1 Hz, 2H, Ar-H), 7.47 (d, *J* = 8.1 Hz, 2H, Ar-H), 7.20 (s, 2H, NH_2_), 6.71 (s, 1H, NH), 5.27 (s, 1H, CH), 4.10 (q, *J* = 7.6 Hz, 4H, 2×CH_2_), 1.20 (t, *J* = 7.6 Hz, 6H, 2×CH_3_); ^13^C NMR (100 MHz, CDCl_3_) δ 171.01, 166.60, 156.84, 148.85, 145.80, 136.05, 134.24, 133.28, 131.28, 119.64, 118.69, 85.22, 61.30, 50.97, 33.64, 20.19; HRMS of [C_20_H_29_N_6_O_4_Cl + 1]^+^ (*m/z*) 443.1246; Calcd: 443.1253.

*Diethyl 6-amino-4-(4-bromophenyl)-5-cyano-1-(1H-1,2,4-triazol-3-yl)-1,4-dihydropyridine-2,3-dicarboxylate* (**5c**)

Pale yellow solid; ^1^H NMR (400 MHz, CDCl_3_) δ 8.80 (s, 1H, triazole-CH), 7.78 (d, *J* = 8.2 Hz, 2H, Ar-H), 7.72 (s, 2H, NH_2_), 7.49 (d, *J* = 8.3 Hz, 2H, Ar-H), 6.75 (s, 1H, NH), 5.36 (s, 1H, CH), 4.44 (q, *J* = 6.9 Hz, 4H, 2×CH_2_), 1.34 (t, *J* = 7.0 Hz, 6H, 2×CH_3_); ^13^C NMR (100 MHz, CDCl_3_) δ 170.50, 166.31, 159.08, 157.15, 152.30, 147.73, 136.86, 135.76, 133.64, 126.37, 124.14, 120.57, 68.65, 50.91, 29.32, 19.45; HRMS of [C_20_H_19_N_6_O_4_Br + 1]^+^ (*m/z*) 487.0483; Calcd: 487.0465.

*Diethyl 6-amino-5-cyano-4-(3,4-dimethoxyphenyl)-1-(1H-1,2,4-triazol-3-yl)-1,4-dihydropyridine-2,3-dicarboxylate* (**5d**)

White solid; ^1^H NMR (400 MHz, CDCl_3_) δ 8.21 (s, 1H, triazole-CH), 7.61 (d, *J* = 1.8 Hz, 1H, Ar-H), 7.58 (s, 2H, NH_2_), 7.32 (dd, *J* = 8.4, 1.9 Hz, 1H, Ar-H), 6.89 (d, *J* = 8.5 Hz, 1H, Ar-H), 6.43 (s, 1H, NH), 5.16 (s, 1H, CH), 4.41 (q, *J* = 7.5 Hz, 4H, 2×CH_2_), 3.92 (s, 3H, OCH_3_), 3.87 (s, 3H, OCH_3_), 2.23 (t, *J* = 7.1 Hz, 6H, 2×CH_3_); ^13^C NMR (100 MHz, CDCl_3_) δ 172.09, 169.67, 159.18, 154.89, 149.56, 145.42, 139.41, 134.73, 128.23, 124.30, 114.44, 113.60, 111.12, 110.80, 78.43, 62.69, 56.35, 56.10, 33.13, 12.09; HRMS of [C_22_H_24_N_6_O_6_ + 1]^+^ (*m/z*) 469.0998; Calcd: 469.0999.

*Diethyl 6-amino-5-cyano-4-(4-(methylthio)phenyl)-1-(1H-1,2,4-triazol-3-yl)-1,4-dihydropyridine-2,3-dicarboxylate* (**5e**)

White solid; ^1^H NMR (400 MHz, CDCl_3_) δ 8.32 (s, 1H, triazole-CH), 7.75 (d, *J* = 8.5 Hz, 2H, Ar-H), 7.59 (s, 2H, NH_2_), 7.24 (d, *J* = 8.5 Hz, 2H, Ar-H), 6.60 (s, 1H, NH), 5.24 (s, 1H, CH), 4.07 (q, *J* = 6.9 Hz, 4H, 2×CH_2_), 2.48 (s, 3H, SCH_3_), 1.92 (t, *J* = 7.1 Hz, 6H, 2×CH_3_); ^13^C NMR (100 MHz, CDCl_3_) δ 176.32, 171.52, 158.81, 149.45, 144.57, 136.64, 131.04, 127.07, 125.47, 120.76, 114.21, 113.10, 80.19, 61.70, 39.49, 19.82, 14.57; HRMS of [C_21_H_22_N_6_O_4_S + 1]^+^ (*m/z*) 455.0694; Calcd: 455.0697.

*Diethyl 6-amino-5-cyano-4-(4-ethylphenyl)-1-(1H-1,2,4-triazol-3-yl)-1,4-dihydropyridine-2,3-dicarboxylate* (**5f**)

Cream white solid; ^1^H NMR (400 MHz, CDCl_3_) δ 8.22 (s, 1H, triazole-CH), 7.64 (d, *J* = 8.4 Hz, 2H, Ar-H), 7.40 (d, *J* = 8.3 Hz, 2H, Ar-H), 7.27 (s, 2H, NH_2_), 6.73 (s, 1H, NH), 5.39 (s, 1H, CH), 4.43 (q, *J* = 7.0 Hz, 4H, 2×CH_2_), 3.04 (q, *J* = 7.2 Hz, 3H, CH_2_CH_3_), 1.34 (t, *J* = 7.0 Hz, 6H, 2×CH_3_), 1.15 (t, *J* = 7.2 Hz, 3H, CH_2_CH_3_); ^13^C NMR (100 MHz, CDCl_3_) δ 170.51, 166.33, 164.81, 159.09, 152.31, 147.30, 138.96, 136.51, 133.87, 120.57, 120.15, 119.18, 68.66, 50.97, 38.98, 29.42, 19.43, 13.97; HRMS of [C_22_H_24_N_6_O_4_ + 1]^+^ (*m/z*) 437.0779; Calcd: 437.0791.

*Diethyl 6-amino-5-cyano-4-(4-(dimethylamino)phenyl)-1-(1H-1,2,4-triazol-3-yl)-1,4-dihydropyridine-2,3-dicarboxylate* (**5g**)

White solid; ^1^H NMR (400 MHz, DMSO-*d_6_*) δ 8.58 (s, 1H, triazole-CH), 8.02 (s, 2H, NH_2_), 7.83 (d, *J* = 9.0 Hz, 2H, Ar-H), 6.84 (d, *J* = 9.1 Hz, 2H, Ar-H), 6.47 (s, 1H, NH), 5.15 (s, 1H, CH), 4.12 (q, *J* = 7.2 Hz, 4H, 2×CH_2_), 3.11 (s, 6H, N(CH_3_)_2_), 2.11 (t, *J* = 7.1 Hz, 6H, 2×CH_3_); ^13^C NMR (100 MHz, DMSO-*d_6_*) δ 176.02, 172.53, 159.30, 154.78, 149.00, 143.00, 138.60, 134.07, 119.22, 116.75, 116.00, 112.25, 69.10, 61.71, 40.41, 31.54, 14.45; HRMS of [C_22_H_25_N_7_O_4_ + 1]^+^ (*m/z*) 452.0721; Calcd: 452.0726.

*Diethyl 6-amino-4-(3-bromophenyl)-5-cyano-1-(1H-1,2,4-triazol-3-yl)-1,4-dihydropyridine-2,3-dicarboxylate* (**5h**)

White solid; ^1^H NMR (400 MHz, CDCl_3_) δ 8.84 (s, 1H, triazole-CH), 7.77 (s, 1H, Ar-H), 7.53 (d, *J* = 5.0 Hz, 2H, Ar-H), 7.48 (s, 2H, NH_2_), 7.31 (s, 1H, Ar-H), 6.66 (s, 1H, NH), 5.40 (s, 1H, CH), 4.44 (q, *J* = 7.0 Hz, 4H, 2×CH_2_), 1.16 (t, *J* = 7.3 Hz, 6H, 2×CH_3_); ^13^C NMR (100 MHz, CDCl_3_) δ 170.45, 166.27, 164.31, 159.04, 152.43, 151.05, 141.61, 136.12, 130.45, 127.10, 126.89, 119.14, 88.77, 88.41, 68.67, 50.96, 29.31, 19.45; HRMS of [C_20_H_19_N_6_O_4_Br + 1]^+^ (*m/z*) 487.0089; Calcd: 487.0106.

*Diethyl 6-amino-5-cyano-4-(3-methoxyphenyl)-1-(1H-1,2,4-triazol-3-yl)-1,4-dihydropyridine-2,3-dicarboxylate* (**5i**)

Pale yellow solid; ^1^H NMR (400 MHz, CDCl_3_) δ 8.78 (s, 1H, triazole-CH), 7.71 (s, 1H, Ar-H), 7.30 (t, *J* = 7.9 Hz, 2H, Ar-H), 7.22 (s, 2H, NH_2_), 6.87 (d, *J* = 5.2 Hz, 1H, Ar-H), 6.83 (s, 1H, NH), 5.30 (s, 1H, CH), 3.80 (q, *J* = 7.1 Hz, 4H, 2×CH_2_), 3.74 (s, 3H, OCH_3_), 1.16 (t, *J* = 7.2 Hz, 6H, 2×CH_3_); ^13^C NMR (100 MHz, CDCl_3_) δ 173.45, 170.61, 164.61, 159.20, 157.09, 152.30, 150.00, 143.94, 135.15, 124.28, 123.29, 118.18, 117.29, 104.60, 61.02, 60.29, 58.98, 50.98, 13.90; HRMS of [C_21_H_22_N_6_O_5_ + 1]^+^ (*m/z*) 439.0764; Calcd: 439.0777.

*Diethyl 6-amino-5-cyano-1-(1H-1,2,4-triazol-3-yl)-4-(2,4,5-trimethoxyphenyl)-1,4-dihydropyridine-2,3-dicarboxylate* (**5j**)

White solid; ^1^H NMR (400 MHz, CDCl_3_) δ 8.70 (s, 1H, triazole-CH), 8.22 (s, 1H, Ar-H), 7.86 (s, 2H, NH_2_), 6.83 (s, 1H, NH), 6.48 (s, 1H, Ar-H), 5.30 (s, 1H, CH), 4.48 (q, *J* = 7.1 Hz, 4H, 2×CH_2_), 4.02 (s, 3H, OCH_3_), 3.93 (s, 6H, 2×OCH_3_), 2.19 (t, *J* = 7.1 Hz, 6H, 2×CH_3_); ^13^C NMR (100 MHz, CDCl_3_) δ 172.29, 169.25, 156.91, 156.55, 152.50, 143.60, 135.06, 131.27, 122.97, 115.35, 114.48, 112.23, 109.20, 95.52, 75.61, 64.50, 56.50, 56.37, 56.30, 31.34, 16.15; HRMS of [C_23_H_26_N_6_O_7_ + 1]^+^ (*m/z*) 499.0644; Calcd: 499.0665.

*Diethyl 6-amino-5-cyano-4-(2-methoxyphenyl)-1-(1H-1,2,4-triazol-3-yl)-1,4-dihydropyridine-2,3-dicarboxylate* (**5k**)

White solid; ^1^H NMR (400 MHz, DMSO-*d_6_*) δ 8.40 (s, 1H, triazole-CH), 7.98 (d, *J* = 8.7 Hz, 1H, Ar-H), 7.48 (d, *J* = 8.5 Hz, 1H, Ar-H), 7.19 (d, *J* = 8.8 Hz, 1H, Ar-H), 7.11 (d, *J* = 8.5 Hz, 1H, Ar-H), 6.72 (s, 2H, NH_2_), 6.64 (s, 1H, NH), 5.28 (s, 1H, CH), 4.43 (q, *J* = 7.0 Hz, 4H, 2×CH_2_), 3.89 (s, 3H, OCH_3_), 1.35 (t, *J* = 7.0 Hz, 6H, 2×CH_3_); ^13^C NMR (100 MHz, DMSO-*d_6_*) δ 172.97, 171.39, 165.94, 164.84, 160.94, 153.99, 133.85, 130.59, 130.31, 124.60, 115.68, 114.55, 114.49, 83.49, 63.75, 55.80, 46.23, 31.15, 21.39; HRMS of [C_21_H_22_N_6_O_5_ + 1]^+^ (*m/z*) 439.1511; Calcd: 439.1509.

*Diethyl 6-amino-5-cyano-4-(p-tolyl)-1-(1H-1,2,4-triazol-3-yl)-1,4-dihydropyridine-2,3-dicarboxylate* (**5l**)

White solid; ^1^H NMR (400 MHz, DMSO-*d_6_*) δ 8.68 (s, 1H, triazole-CH), 7.92 (d, *J* = 8.9 Hz, 2H, Ar-H), 7.54 (d, *J* = 8.8 Hz, 2H, Ar-H), 7.26 (s, 2H, NH_2_), 6.95 (s, 1H, NH), 5.53 (s, 1H, CH), 4.15 (q, *J* = 7.2 Hz, 4H, 2×CH_2_), 2.09 (s, 3H, CH_3_), 1.37 (t, *J* = 7.0 Hz, 6H, 2×CH_3_); ^13^C NMR (100 MHz, DMSO-*d_6_*) δ 175.96, 169.13, 162.33, 158.14, 152.61, 133.27, 126.78, 124.37, 120.75, 119.30, 114.59, 89.33, 62.16, 46.23, 31.16, 24.75, 9.11; HRMS of [C_21_H_22_N_6_O_4_ + 1]^+^ (*m/z*) 423.0108; Calcd: 423.0098.

# Supplementary Figures


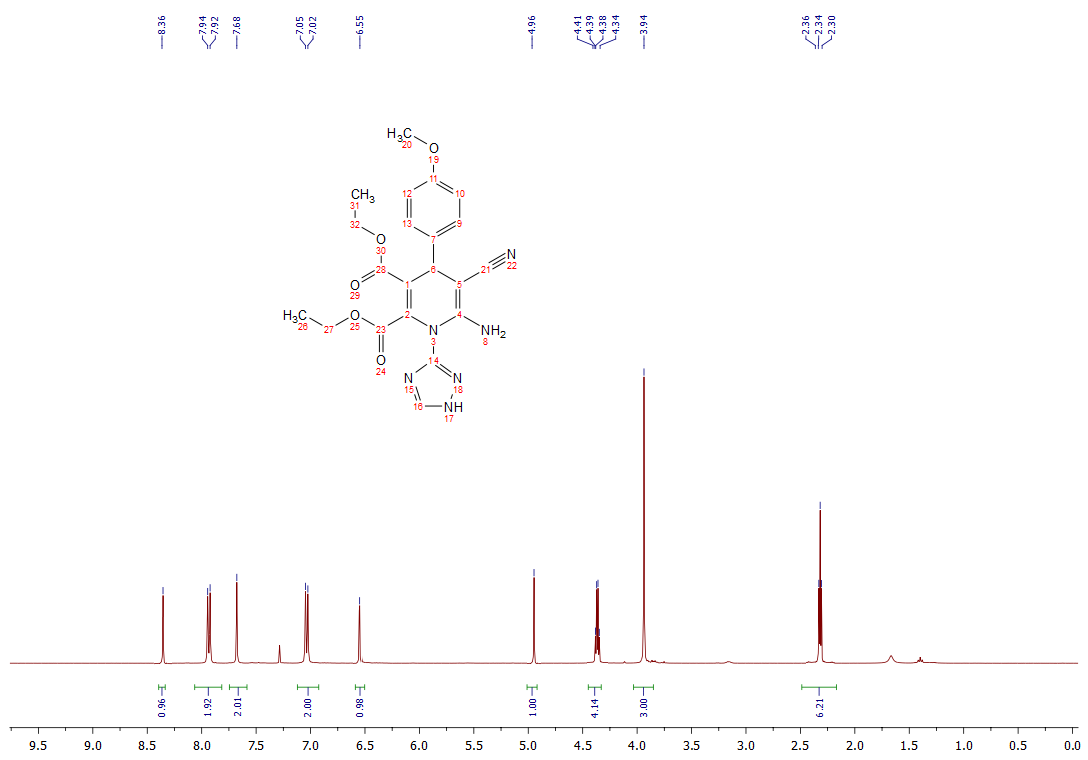


**Figure 1.** ^1^H-NMR spectra of compound **5a**


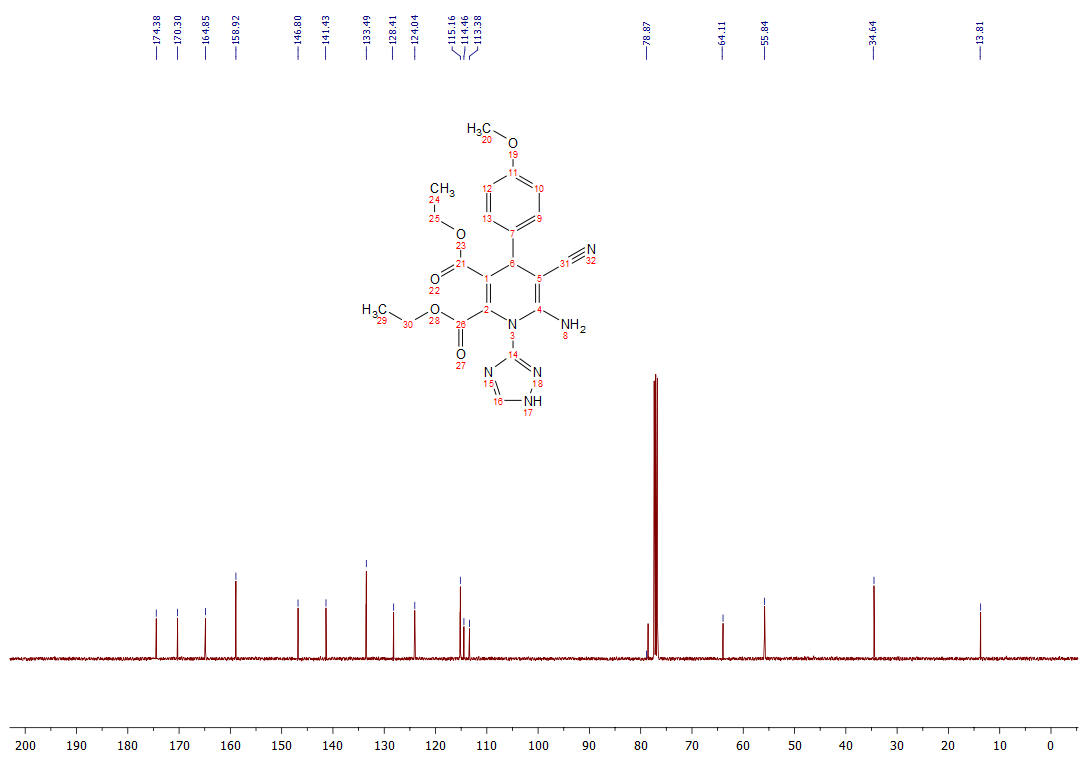


**Figure 2.** ^13^C-NMR spectra of compound **5a**


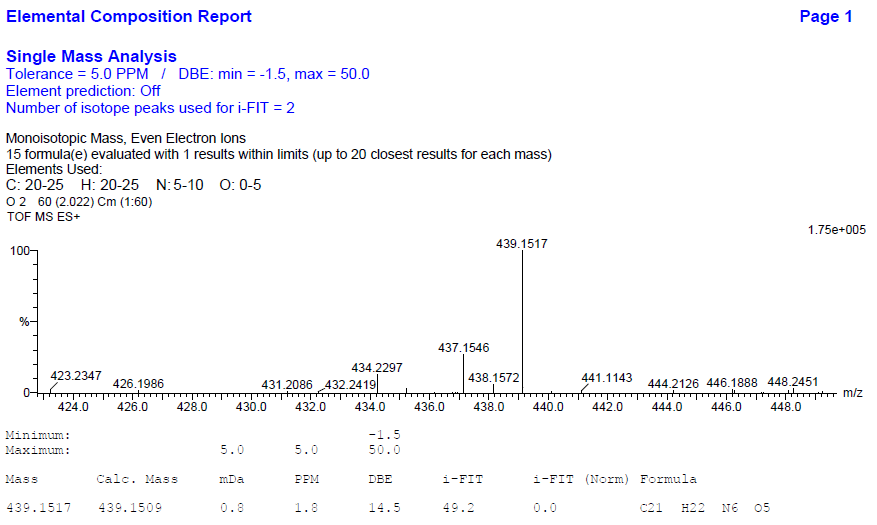


**Figure 3.** HRMS-spectra of compound **5a**


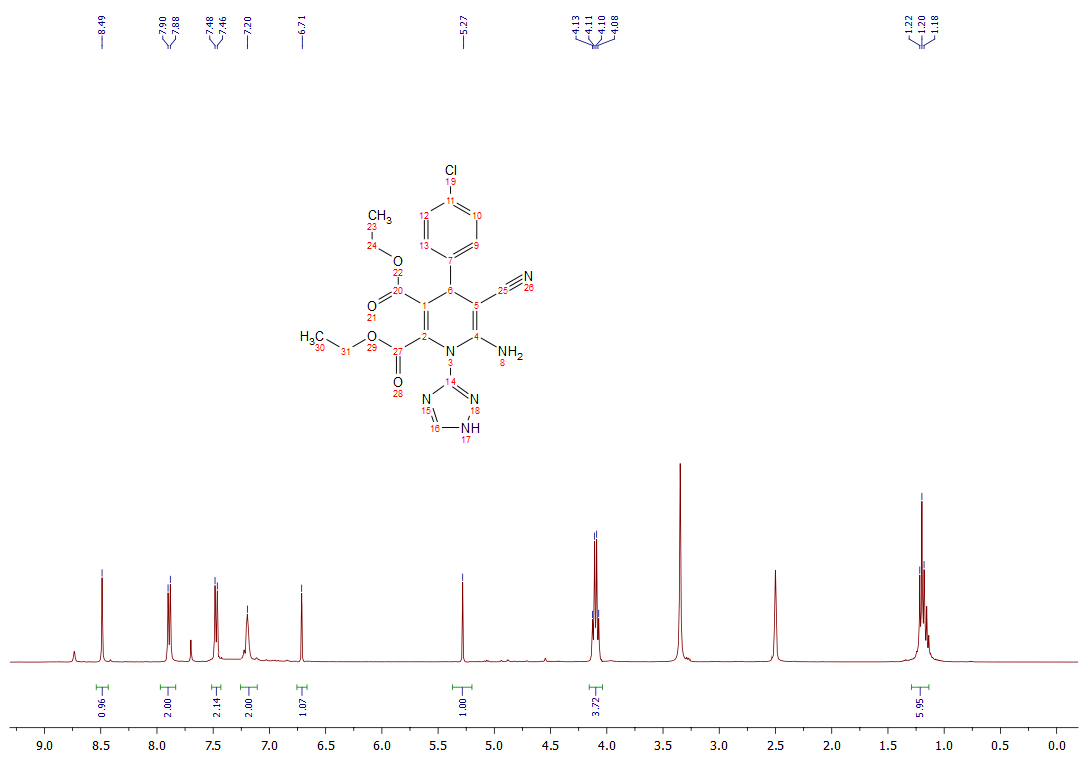


**Figure 4.** ^1^H-NMR spectra of compound **5b**


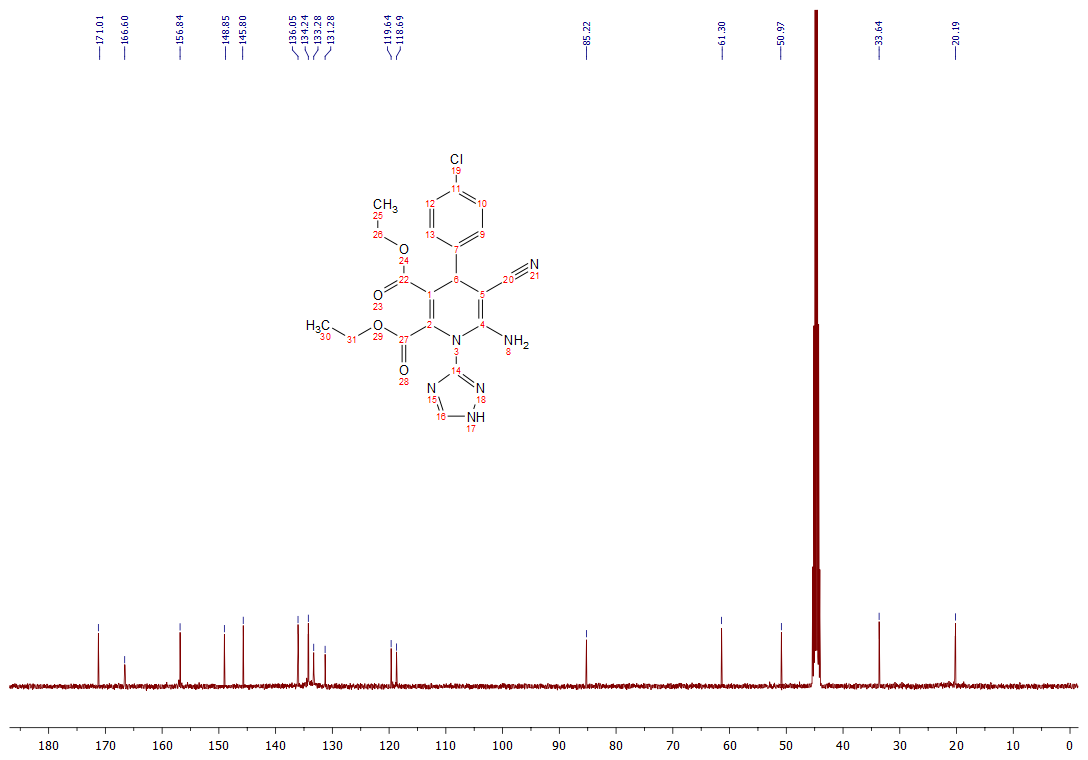


**Figure 5.** ^13^C-NMR spectra of compound **5b**


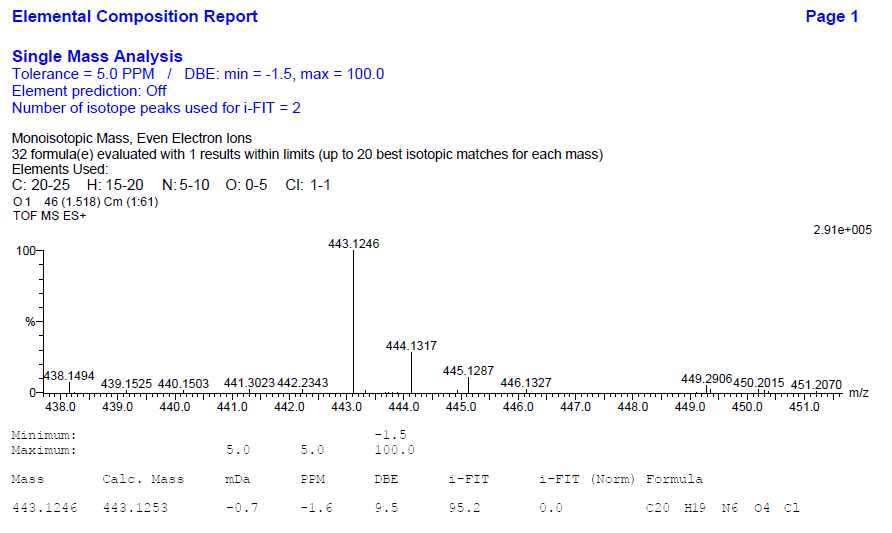


**Figure 6.** HRMS-spectra of compound **5b**


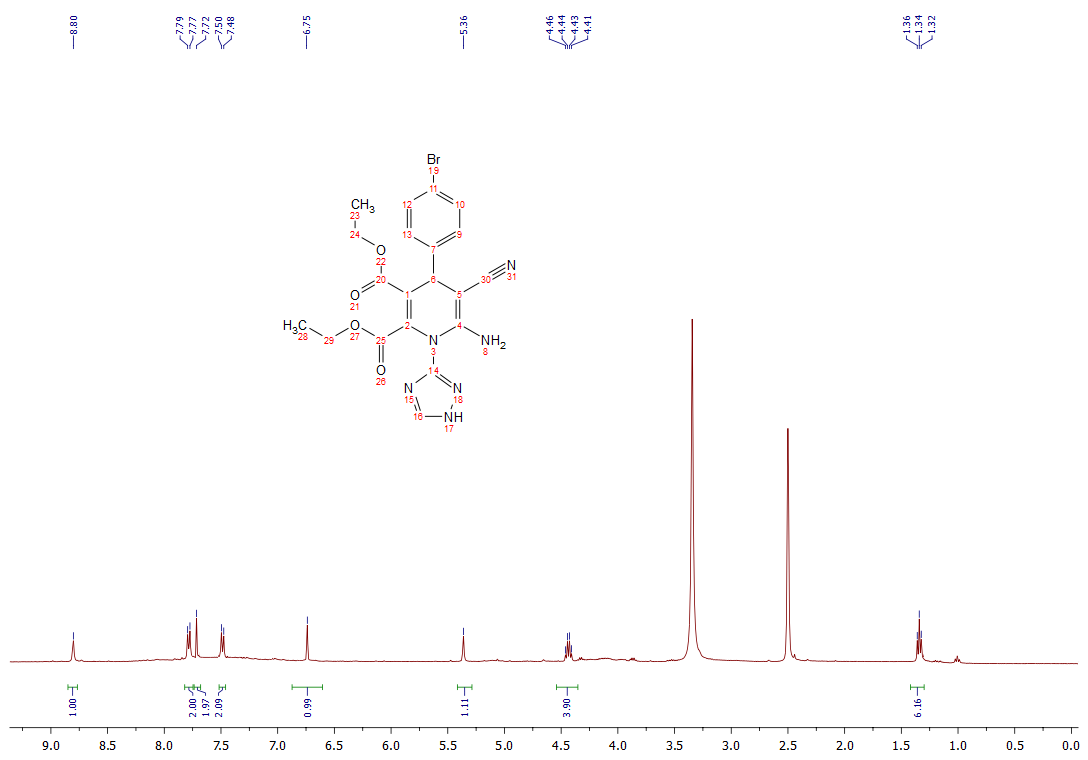


**Figure 7.** ^1^H-NMR spectra of compound **5c**


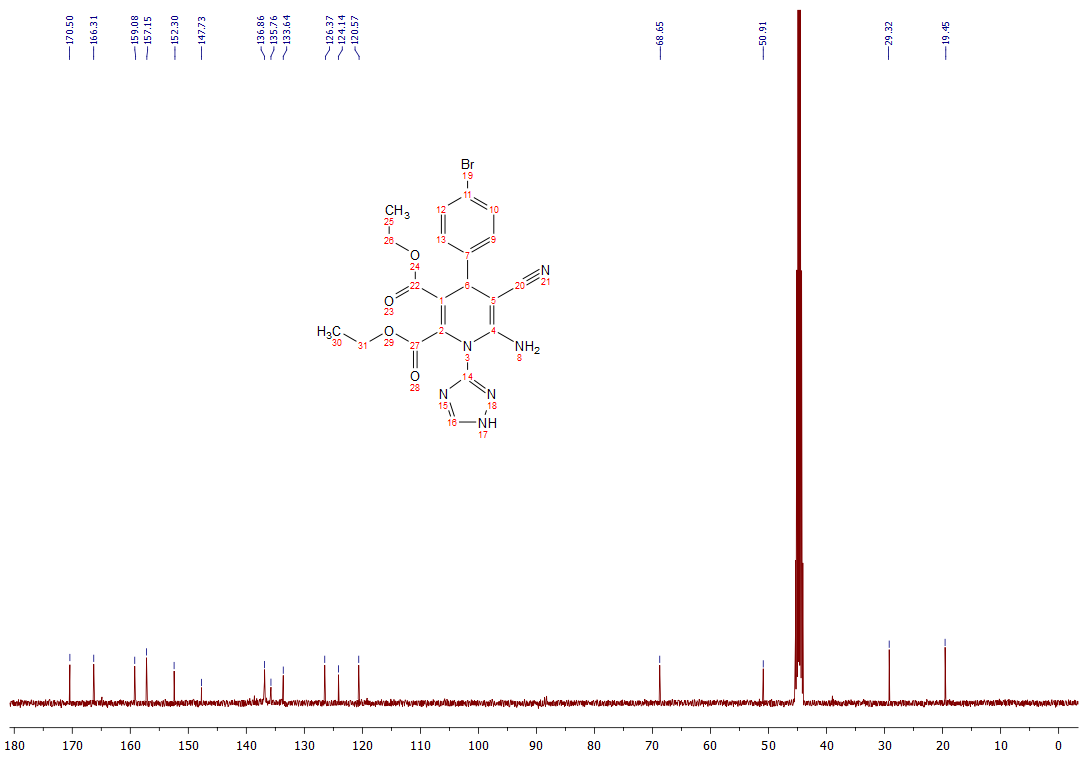


**Figure 8.** ^13^C-NMR spectra of compound **5c**


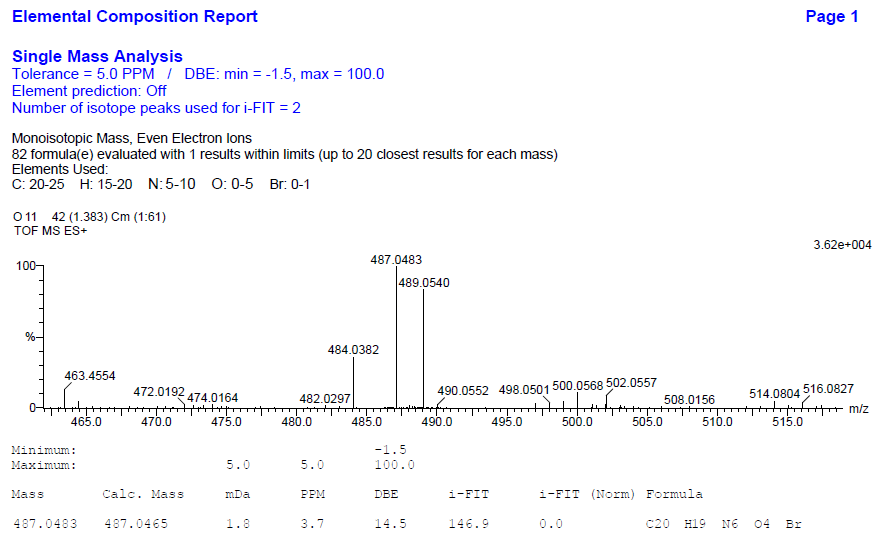


**Figure 9.** HRMS-spectra of compound **5c**


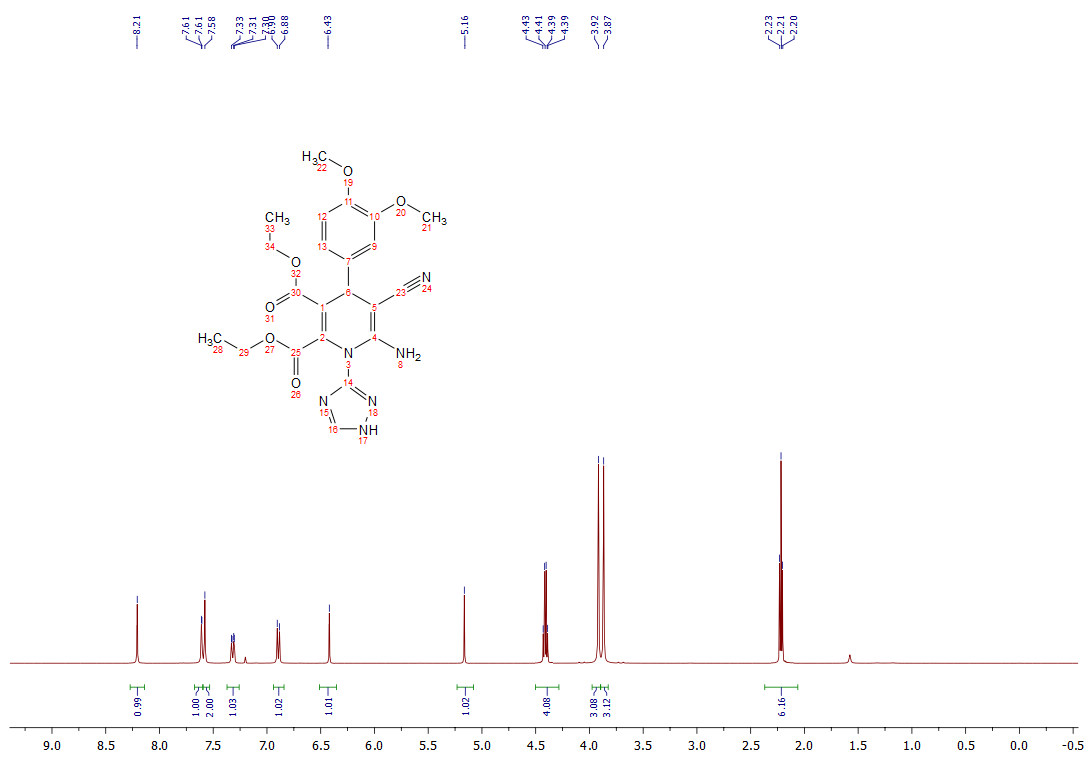


**Figure 10.** ^1^H-NMR spectra of compound **5d**


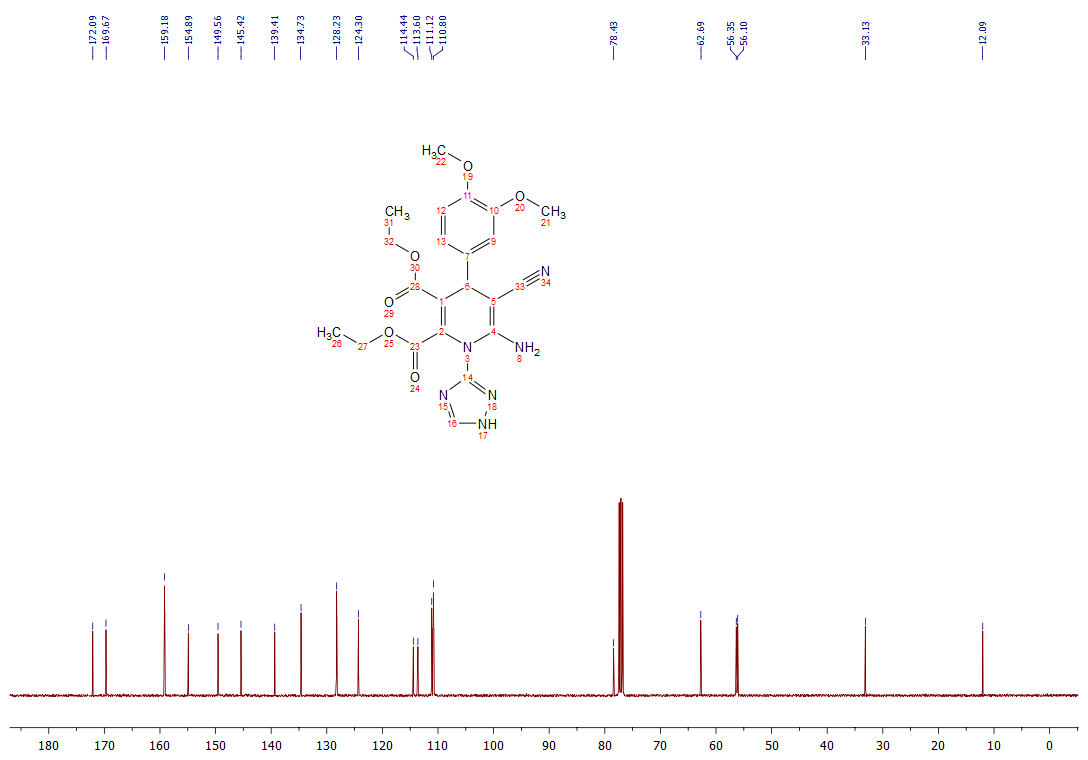


**Figure 11.** ^13^C-NMR spectra of compound **5d**


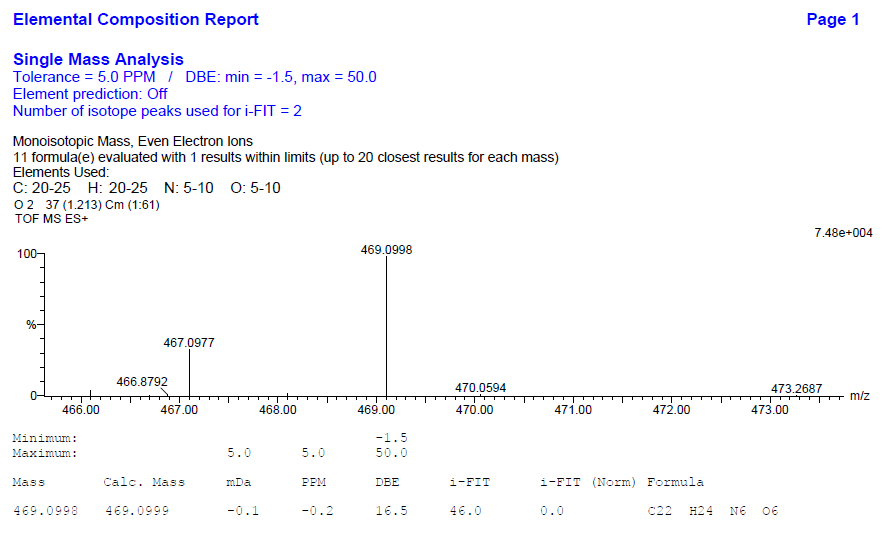


**Figure 12.** HRMS-spectra of compound **5d**


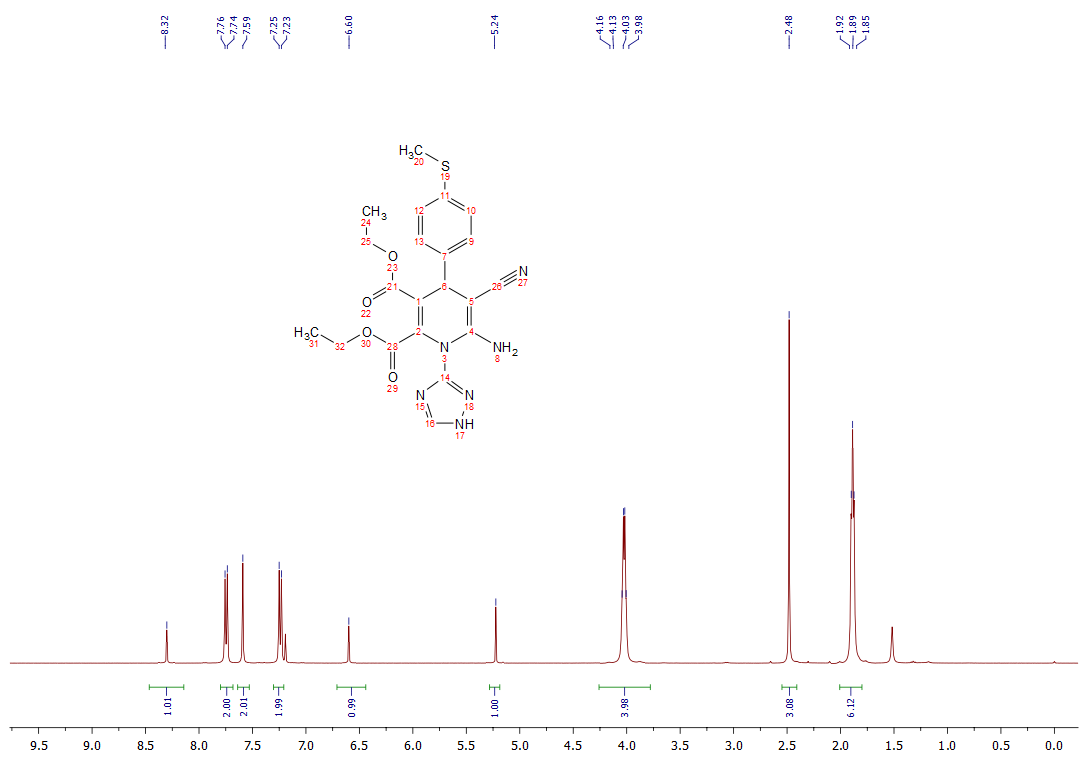


**Figure 13.** ^1^H-NMR spectra of compound **5e**


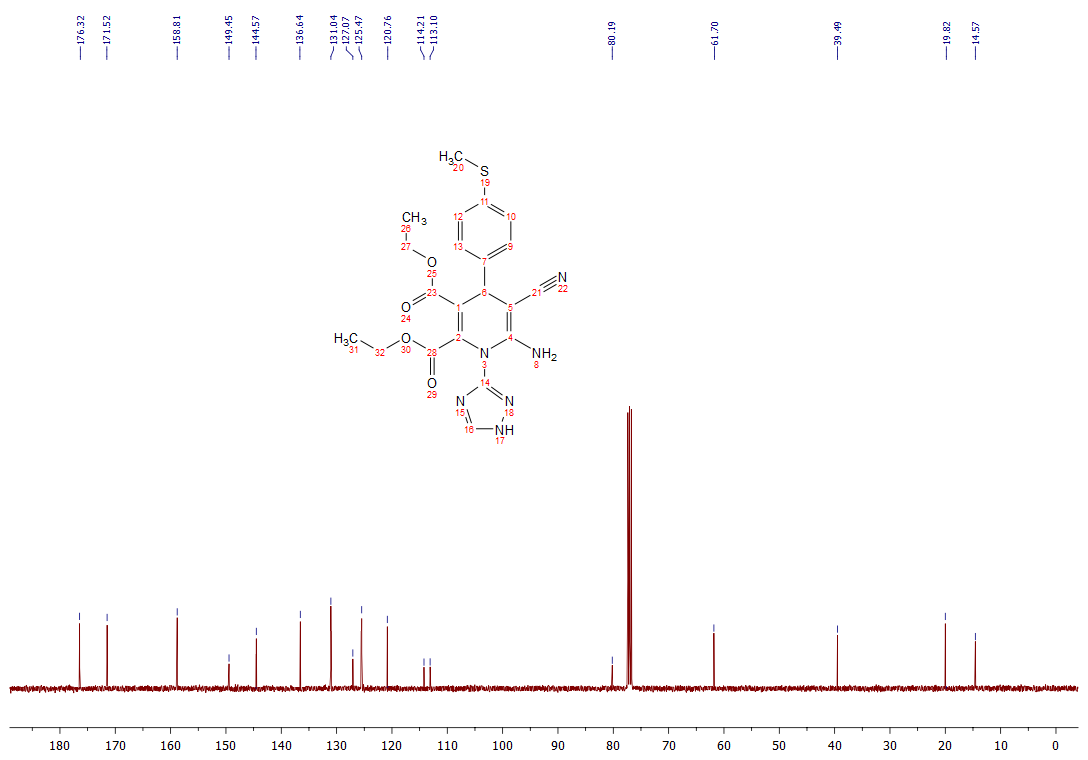


**Figure 14.** ^13^C-NMR spectra of compound **5e**


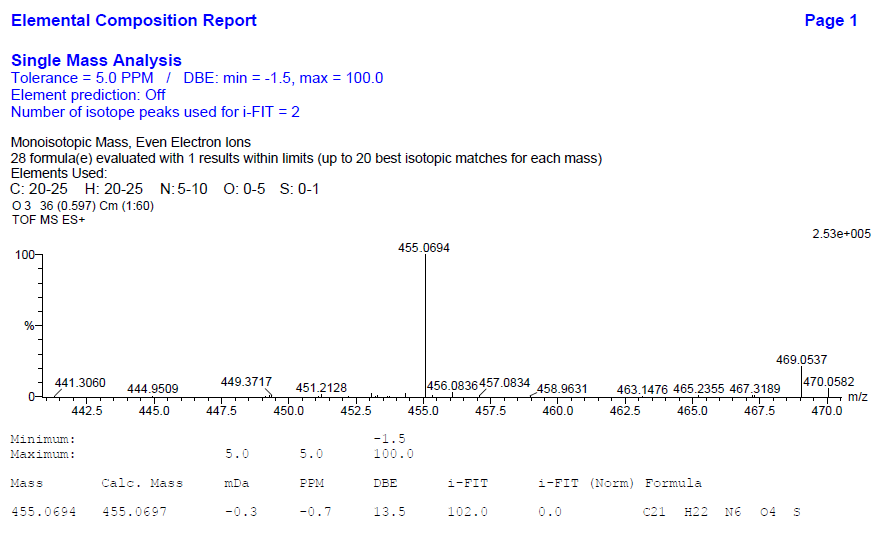


**Figure 15.** HRMS-spectra of compound **5e**


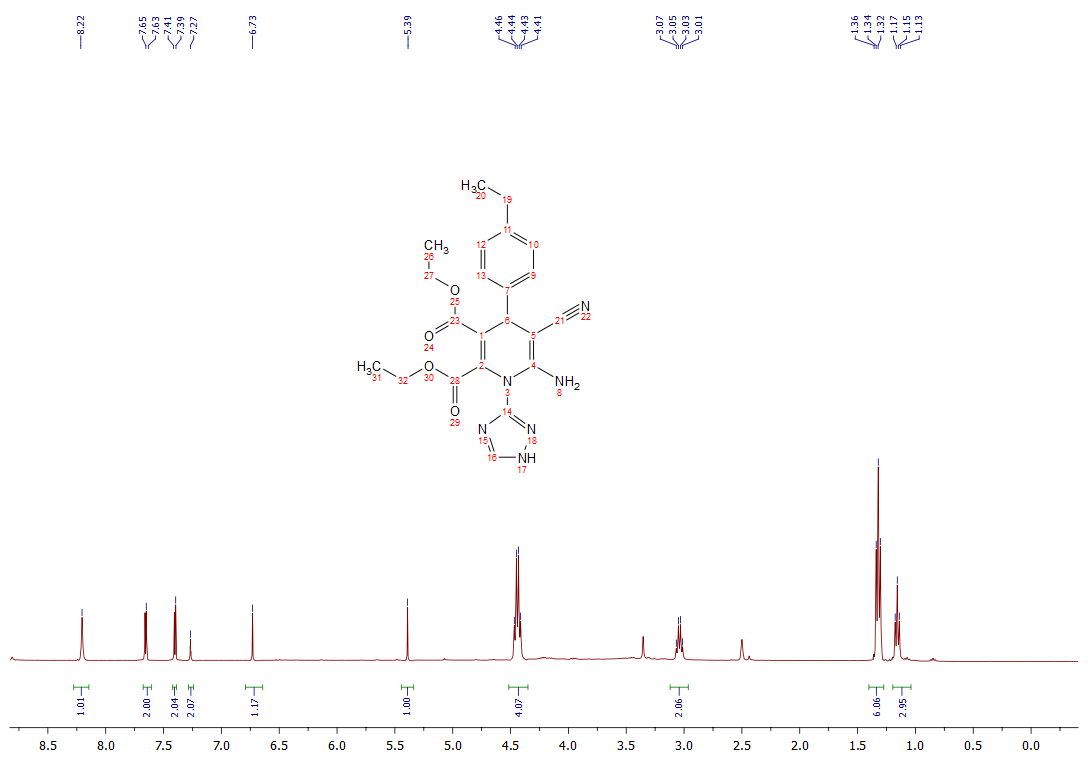


**Figure 16.** ^1^H-NMR spectra of compound **5f**


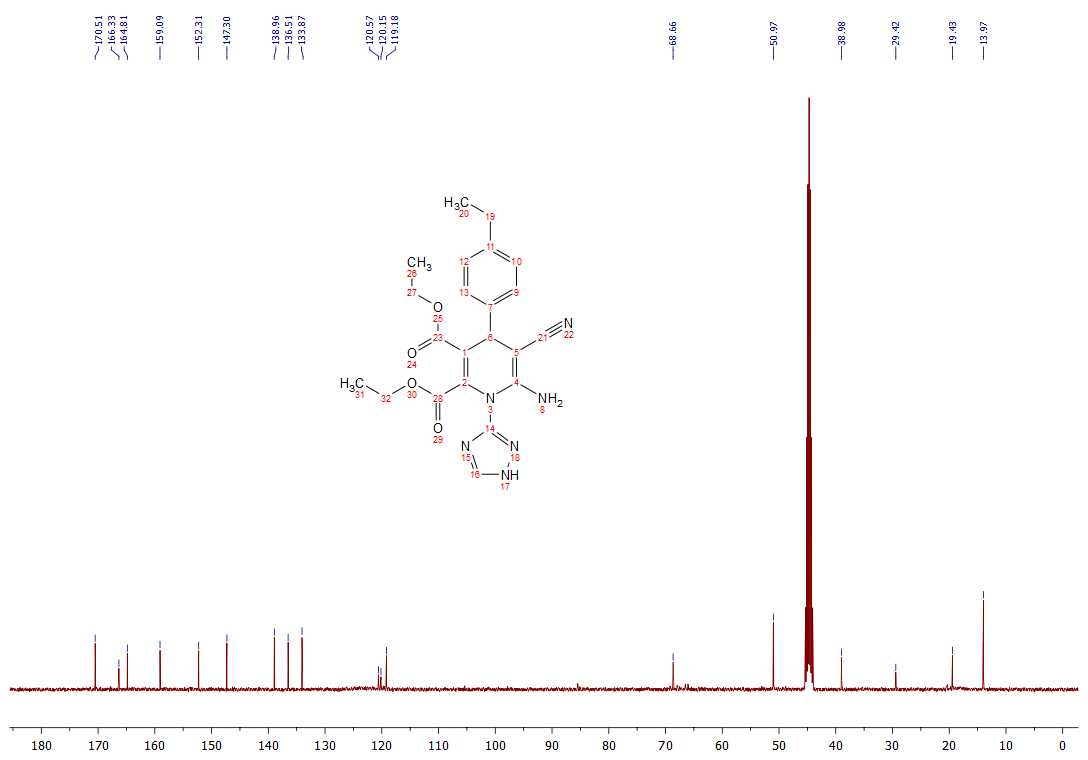


**Figure 17.** ^13^C-NMR spectra of compound **5f**


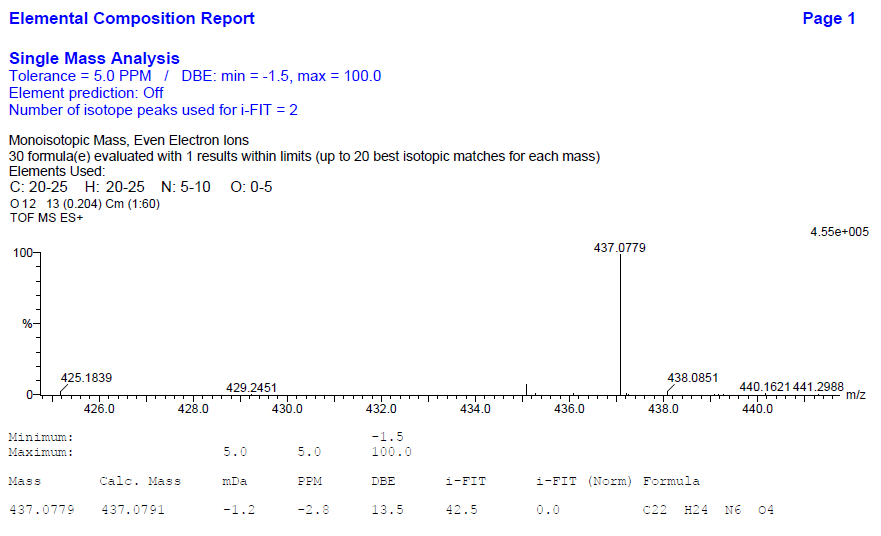


**Figure 18.** HRMS-spectra of compound **5f**


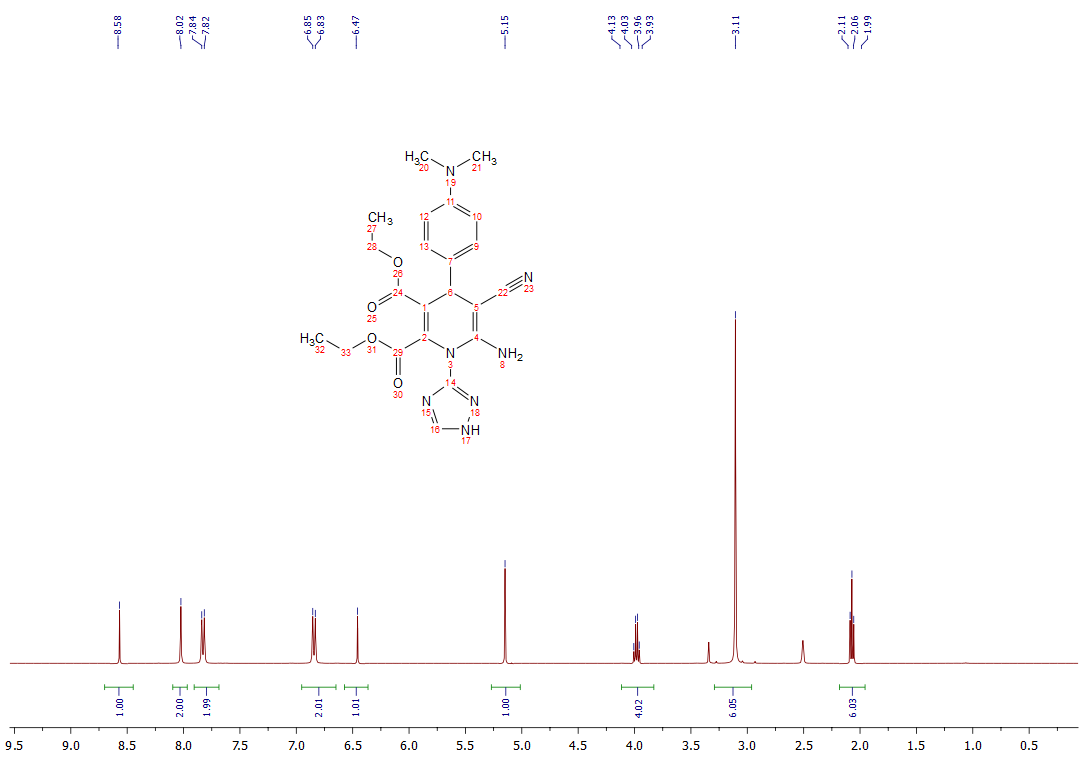


**Figure 19.** ^1^H-NMR spectra of compound **5g**


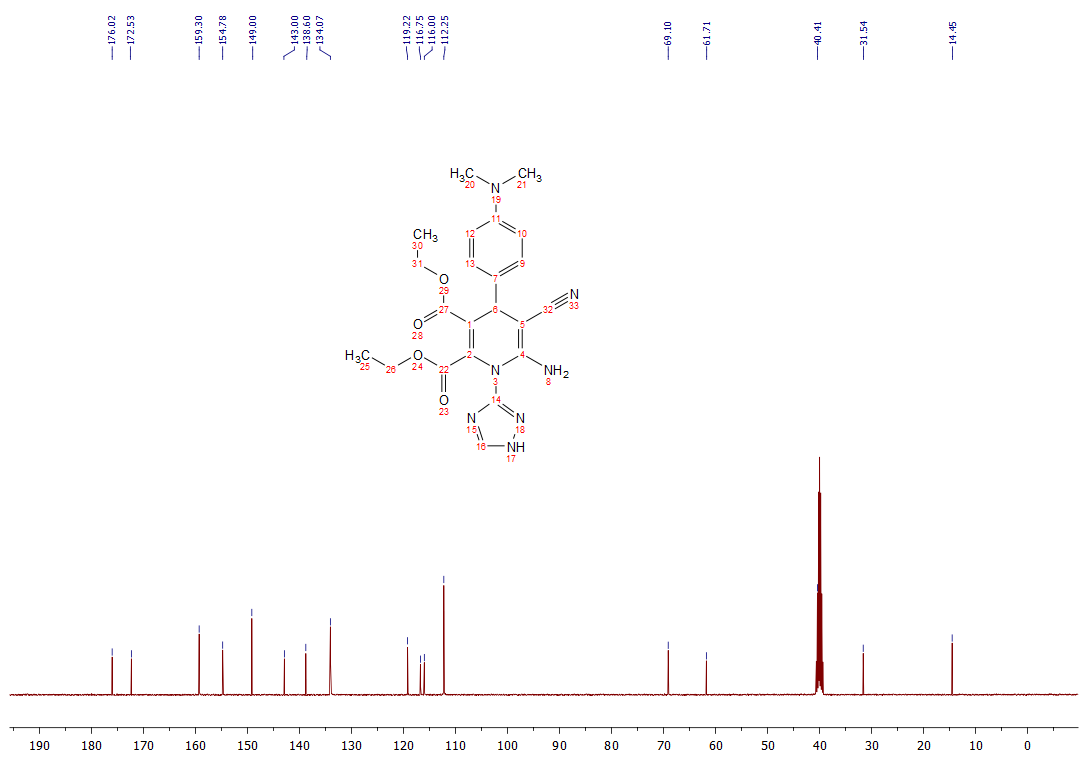


**Figure 20.** ^13^C-NMR spectra of compound **5g**


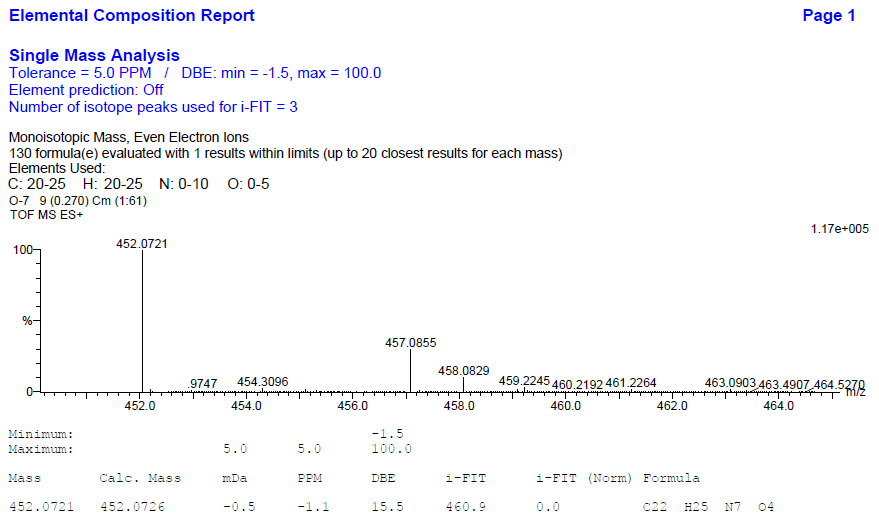


**Figure 21.** HRMS-spectra of compound **5g**


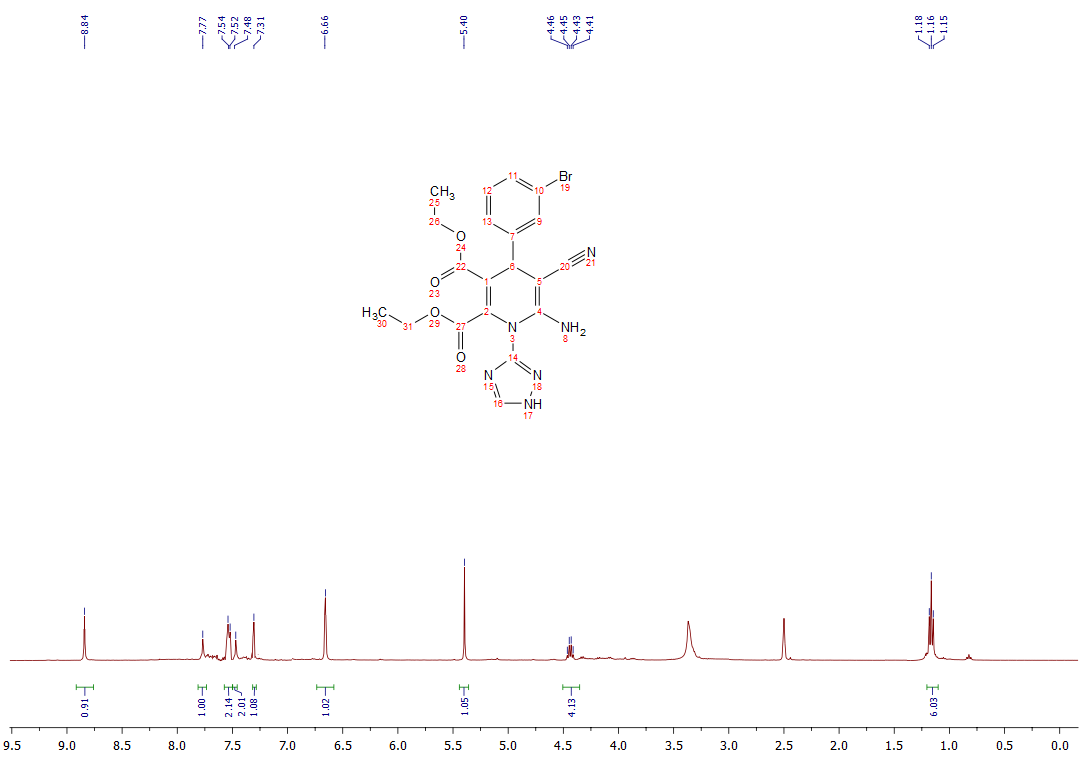


**Figure 22.** ^1^H-NMR spectra of compound **5h**


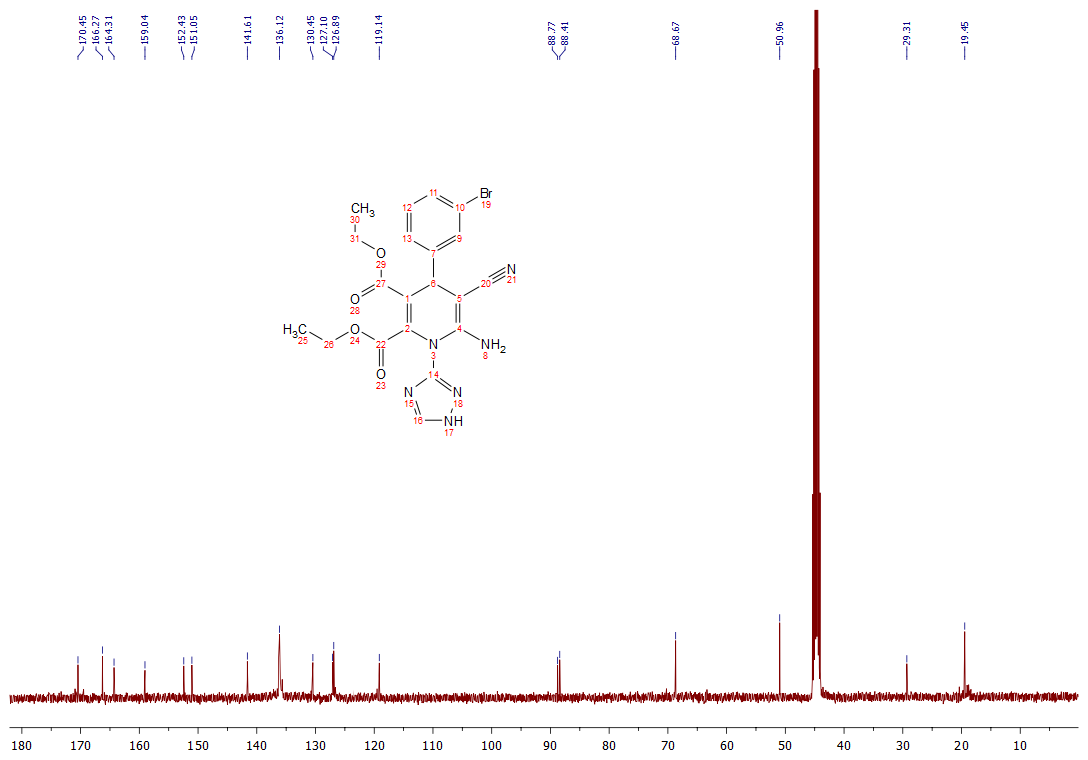


**Figure 23.** ^13^C-NMR spectra of compound **5h**


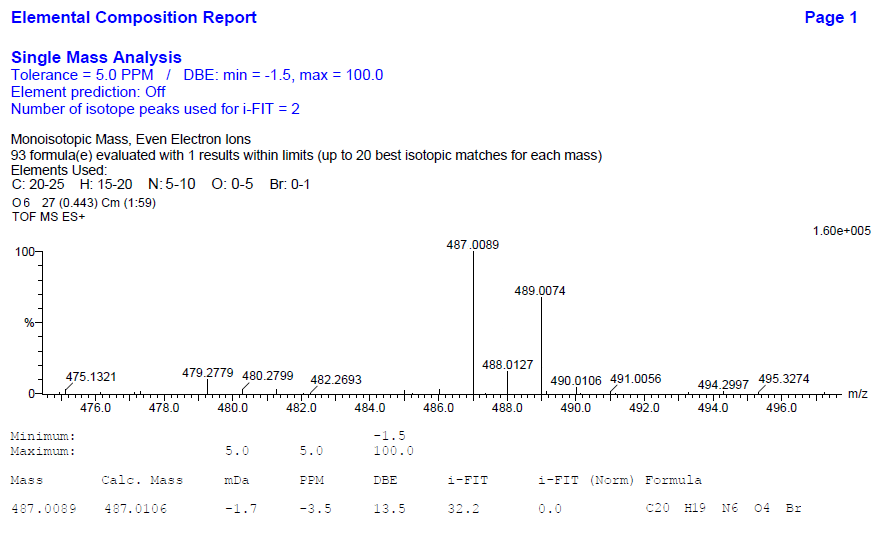


**Figure 24.** HRMS-spectra of compound **5h**


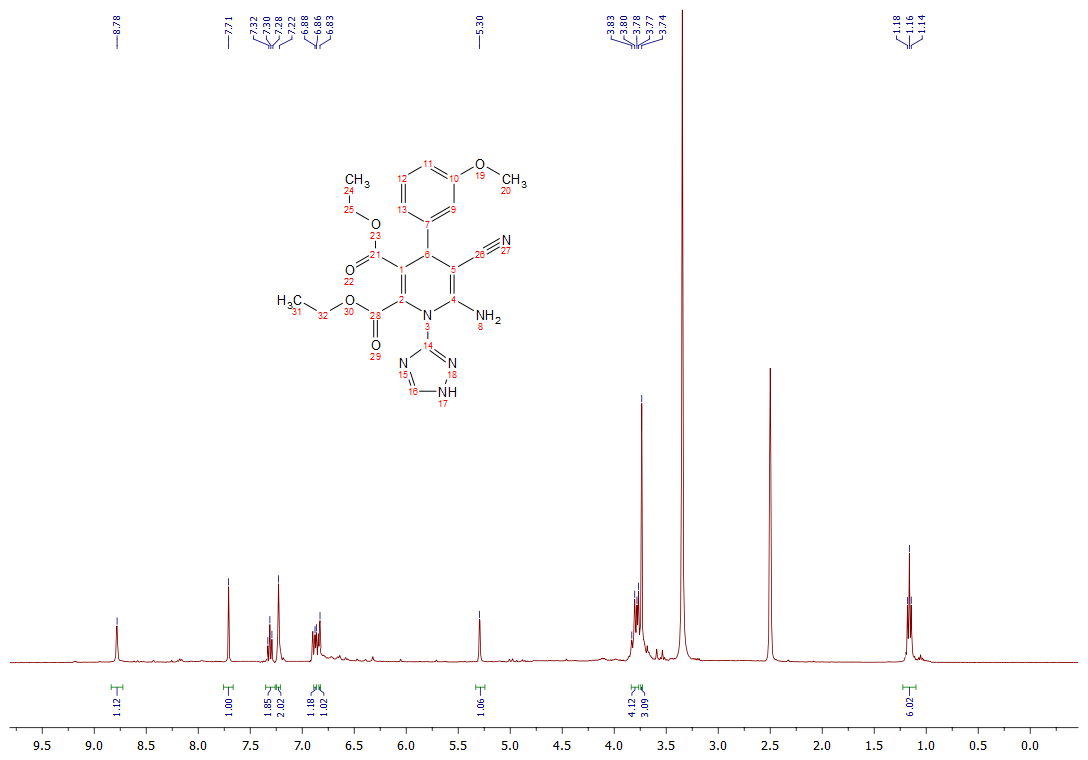


**Figure 25.** ^1^H-NMR spectra of compound **5i**


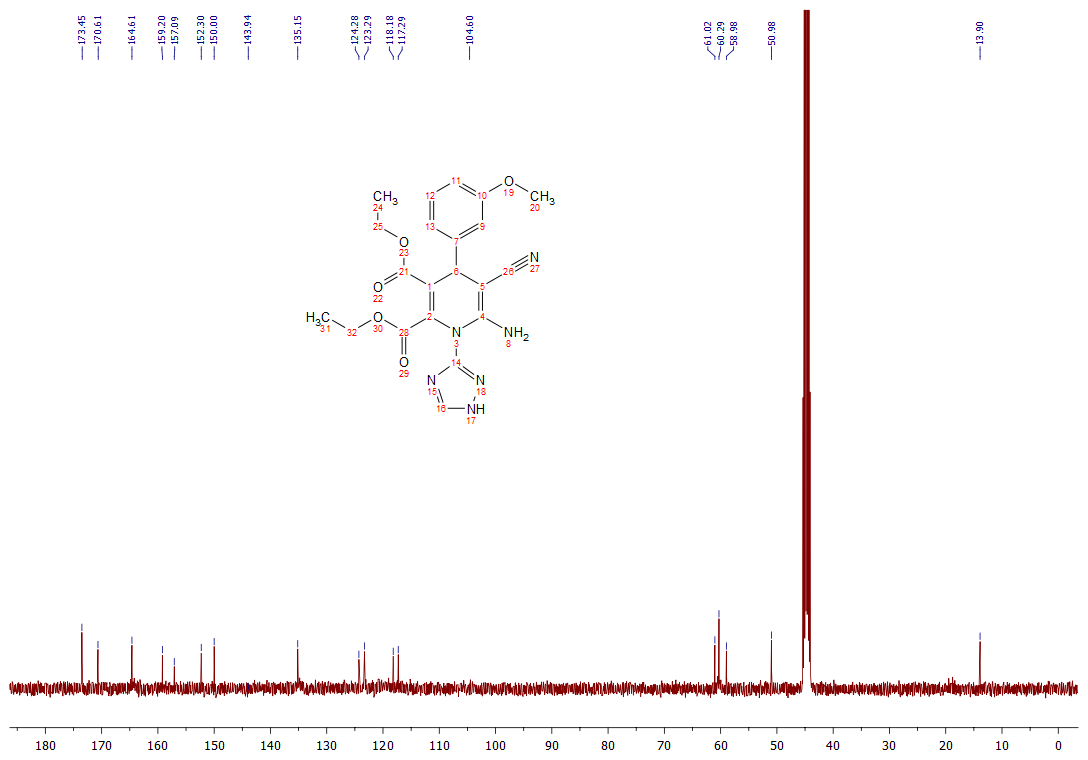


**Figure 26.** ^13^C-NMR spectra of compound **5i**


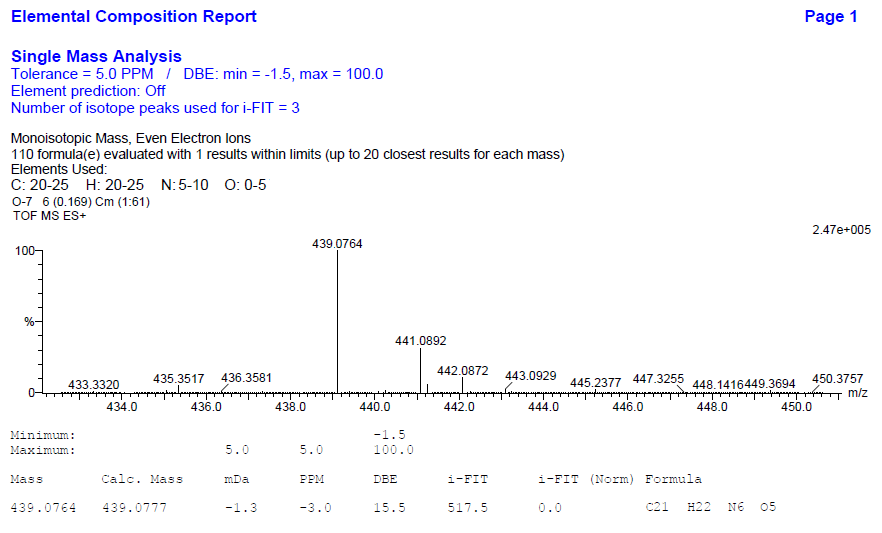


**Figure 27.** HRMS-spectra of compound **5i**


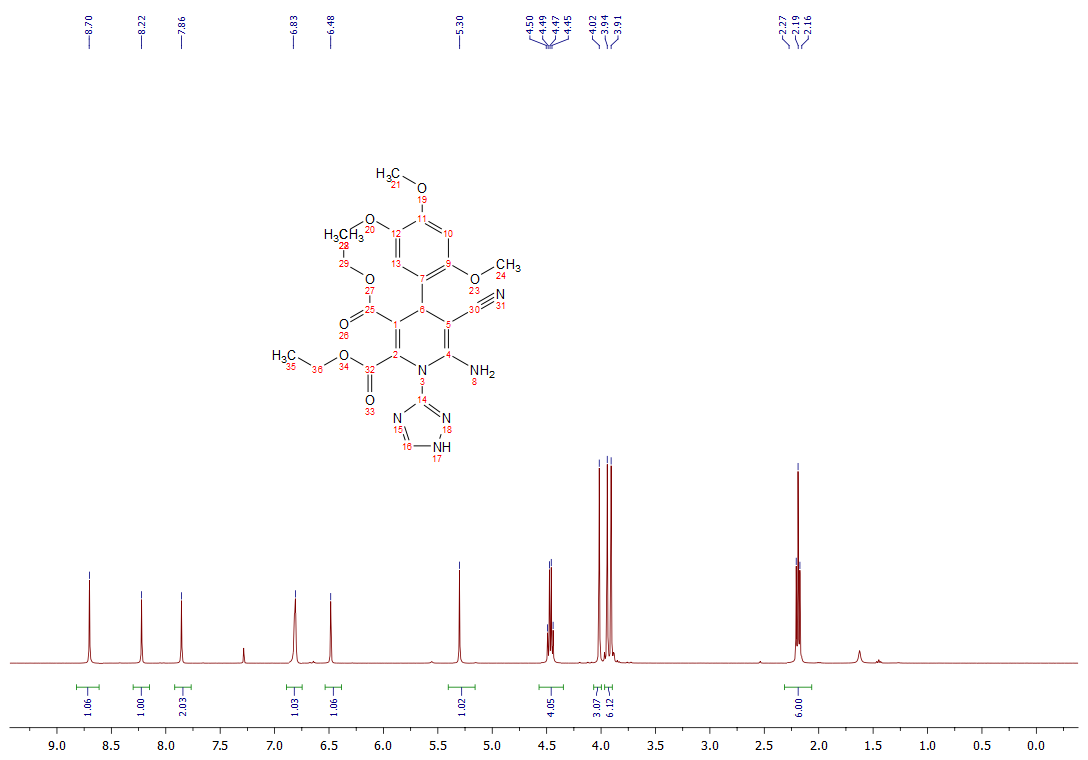


**Figure 28.** ^1^H-NMR spectra of compound **5j**


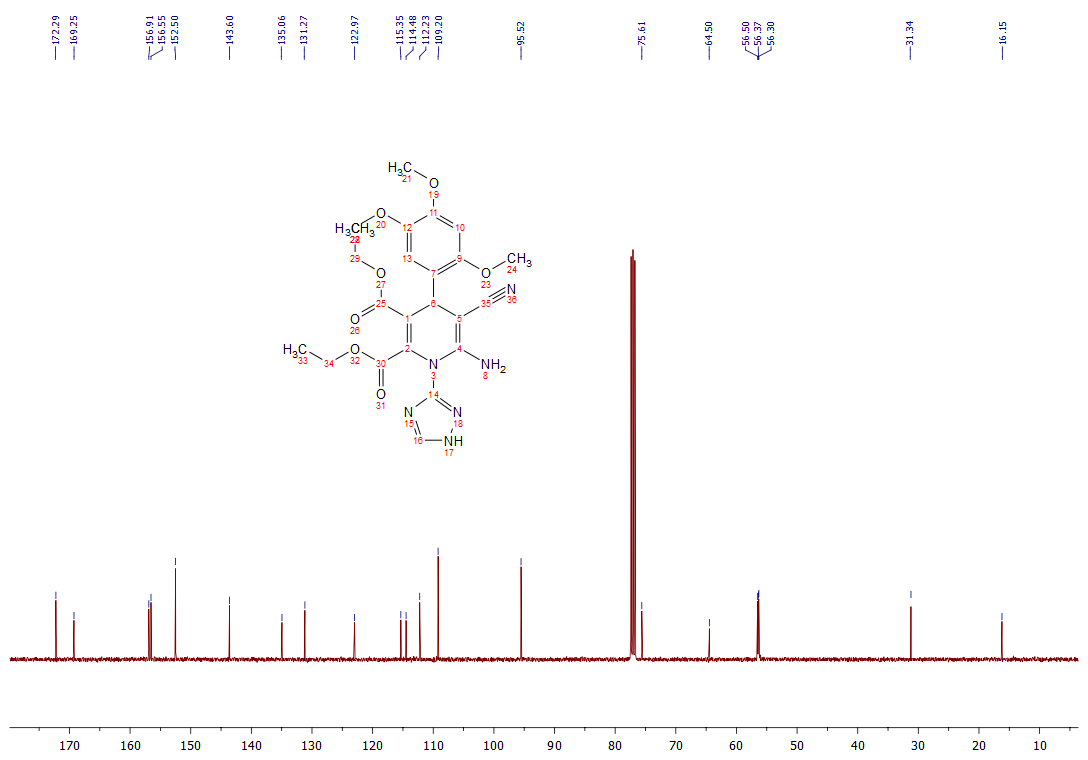


**Figure 29.** ^13^C-NMR spectra of compound **5j**


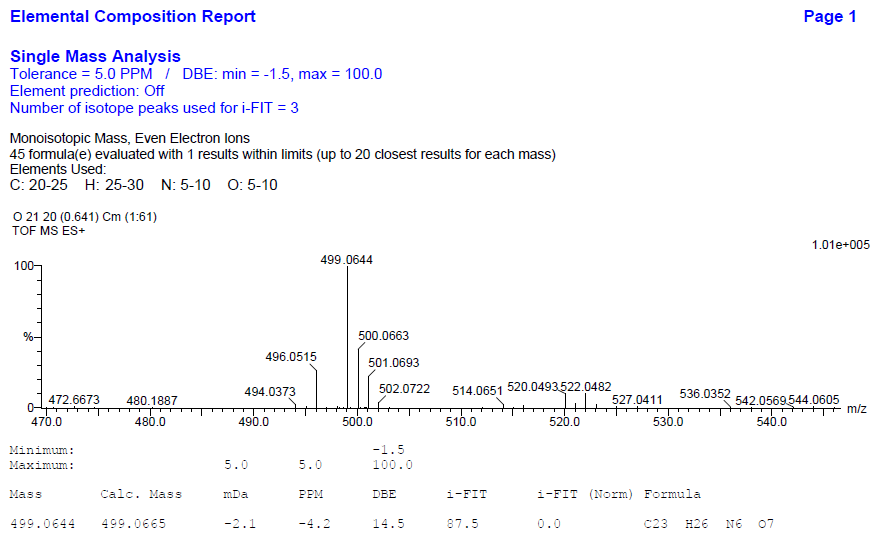


**Figure 30.** HRMS-spectra of compound **5j**


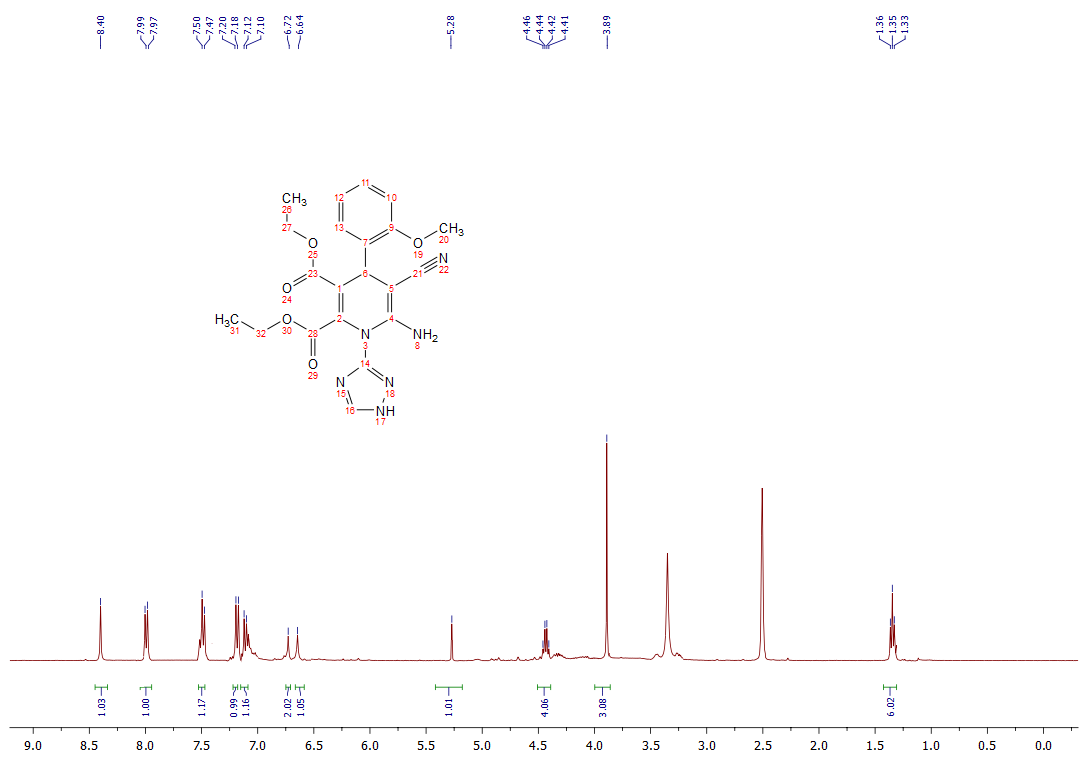


**Figure 31.** ^1^H-NMR spectra of compound **5k**


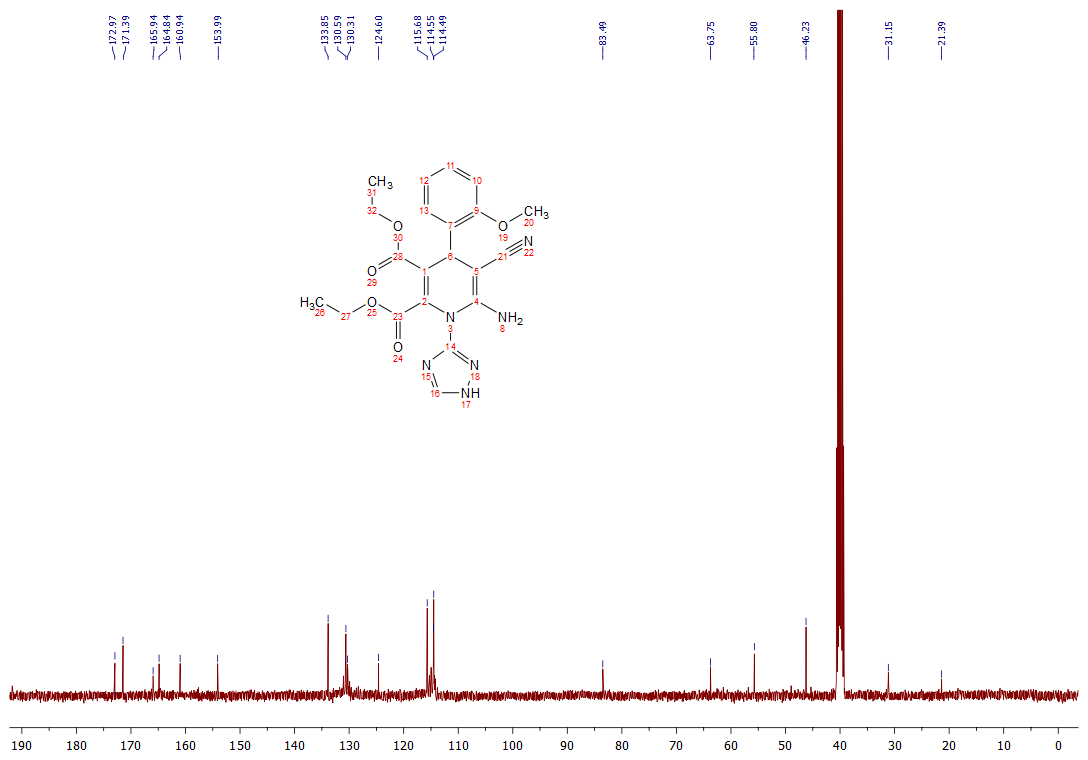


**Figure 32.** ^13^C-NMR spectra of compound **5k**


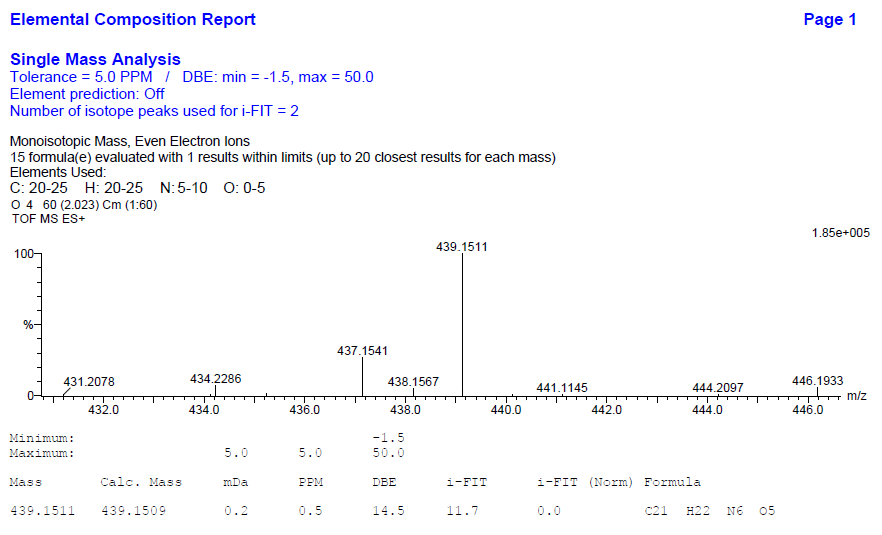


**Figure 33.** HRMS-spectra of compound **5k**


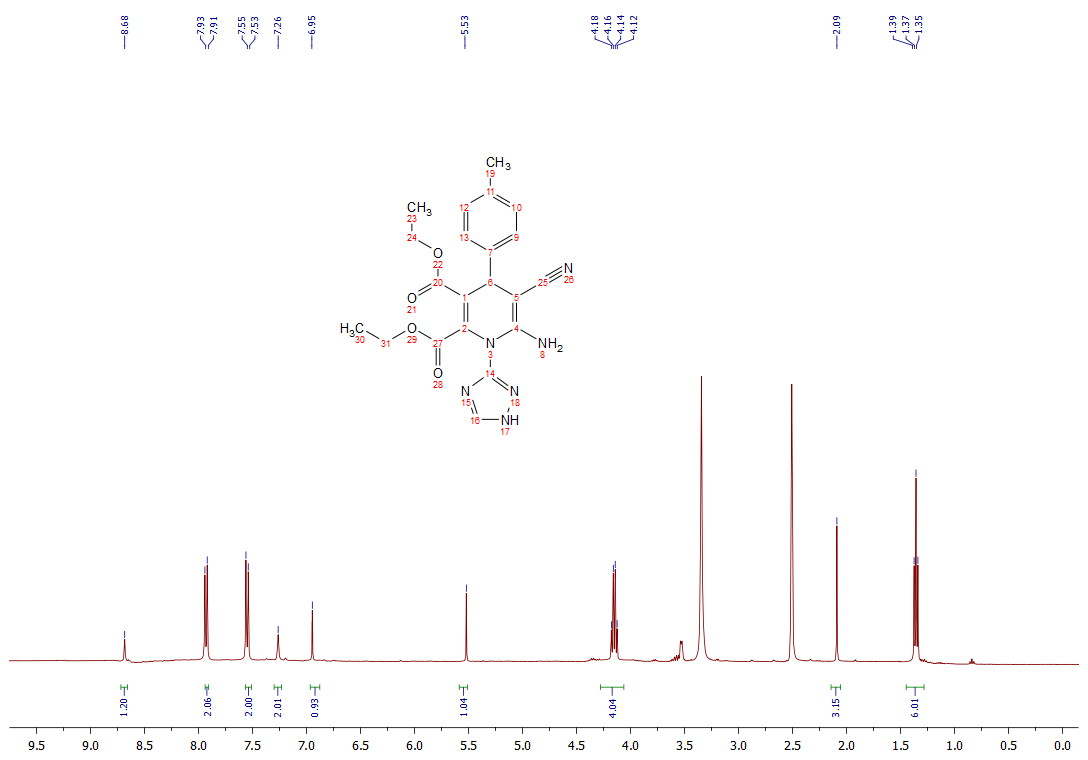


**Figure 34.** ^1^H-NMR spectra of compound **5l**


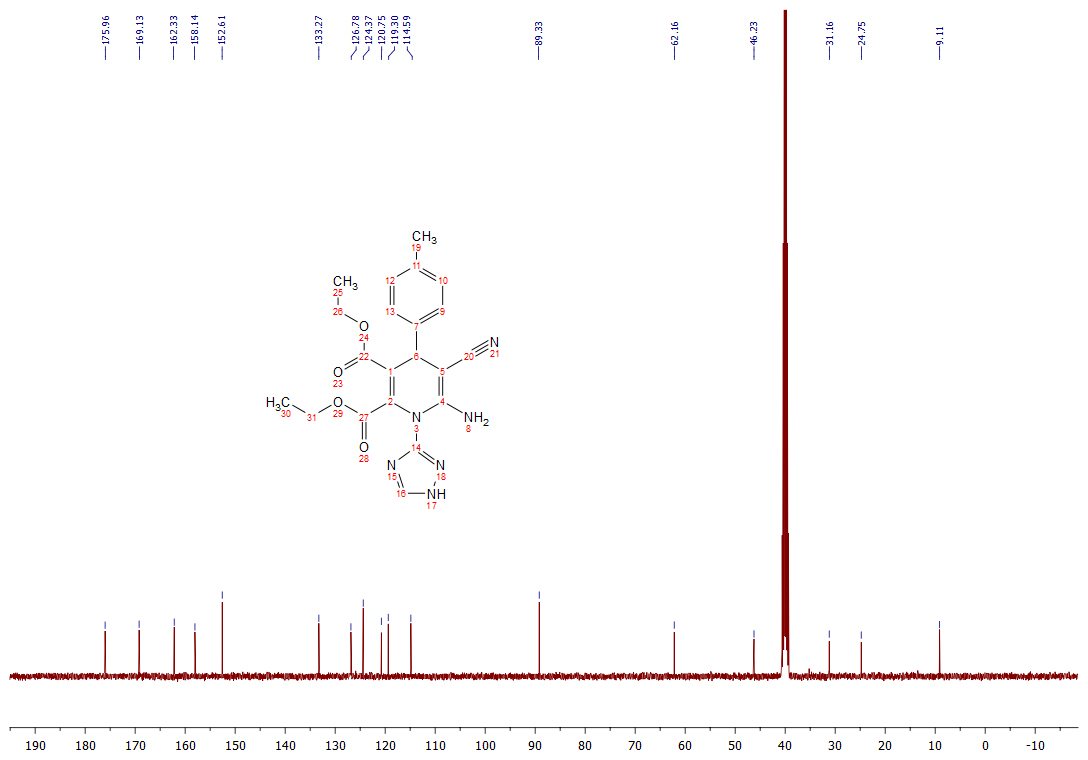


**Figure 35.** ^13^C-NMR spectra of compound **5l**


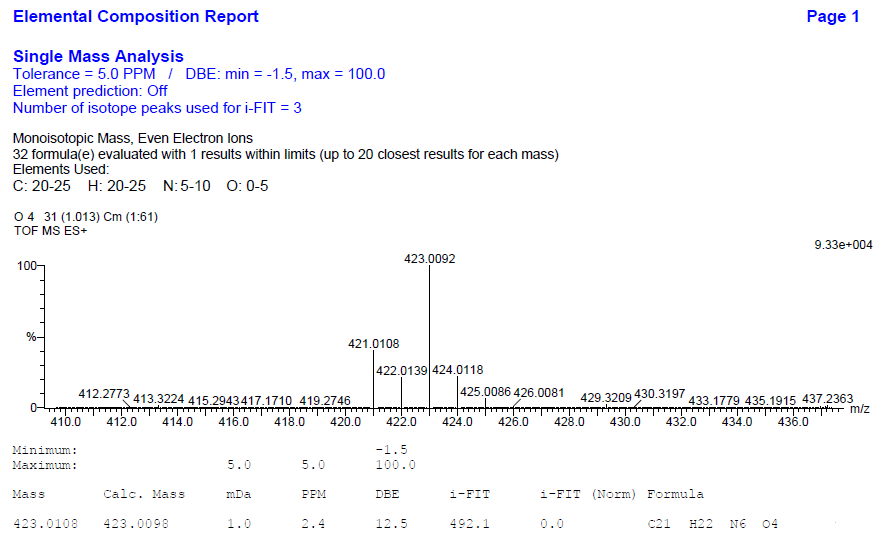


**Figure 36.** HRMS-spectra of compound **5l**
